# Paper Spray Ionization Ion Mobility Mass Spectrometry of Sebum Classifies Biomarker Classes for the Diagnosis of Parkinson’s Disease

**DOI:** 10.1021/jacsau.2c00300

**Published:** 2022-09-07

**Authors:** Depanjan Sarkar, Eleanor Sinclair, Sze Hway Lim, Caitlin Walton-Doyle, Kaneez Jafri, Joy Milne, Johannes P.C. Vissers, Keith Richardson, Drupad K. Trivedi, Monty Silverdale, Perdita Barran

**Affiliations:** † Manchester Institute of Biotechnology, School of Chemistry, The University of Manchester, Princess Street, Manchester M1 7DN, UK; ‡ Department of Neurology, Salford Royal Foundation Trust, Manchester Academic Health Science Centre, University of Manchester, Manchester M13 9NQ, UK; § Waters Corporation, Stamford Avenue, Altrincham Road, Wilmslow SK9 4AX, UK

**Keywords:** Parkinson’s disease, biomarker classes, sebum, paper spray ionization, ion mobility, lipids

## Abstract

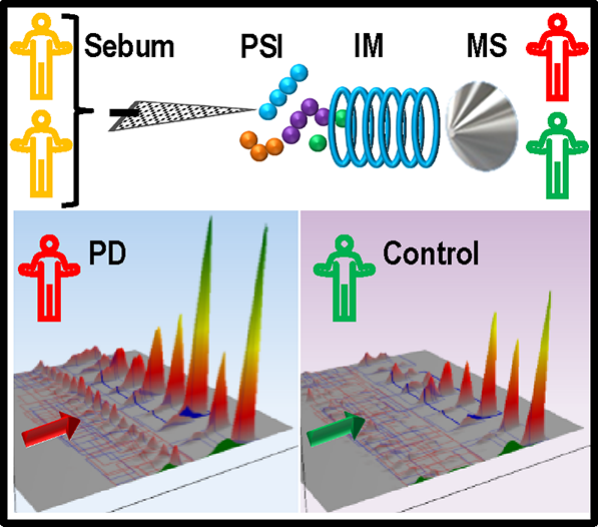

Parkinson’s disease (PD) is the second most common neurodegenerative disorder, and identification of robust biomarkers to complement clinical diagnosis will accelerate treatment options. Here, we demonstrate the use of direct infusion of sebum from skin swabs using paper spray ionization coupled with ion mobility mass spectrometry (PS-IM-MS) to determine the regulation of molecular classes of lipids in sebum that are diagnostic of PD. A PS-IM-MS method for sebum samples that takes 3 min per swab was developed and optimized. The method was applied to skin swabs collected from 150 people and elucidates ∼4200 features from each subject, which were independently analyzed. The data included high molecular weight lipids (>600 Da) that differ significantly in the sebum of people with PD. Putative metabolite annotations of several lipid classes, predominantly triglycerides and larger acyl glycerides, were obtained using accurate mass, tandem mass spectrometry, and collision cross section measurements.

## Introduction

Neurodegenerative diseases are the leading source of disability globally.^1^ According to the 2015 Global Burden of Disease, Injuries, and Risk Factors Study (GBD), Parkinson’s disease (PD) is globally the fastest growing neurological disorder.^1^ PD is also the second most common age-related neurodegenerative disorder with a prevalence of approximately 2% among people aged 65 and over, with motor symptoms including bradykinesia, tremor, rigidity, and postural instability as well as several non-motor symptoms.^2,3^ While some of this increase tracks the demographics of greater life expectancies, this does not account for it entirely. The increased occurrence may also be due to better diagnosis and to environmental factors, particularly in mid social security disability insurance (SSDI) countries. Currently, it is predicted that these numbers are projected to increase globally to over 20 million by 2050.^1^ The increase in PD globally, and its commensurate prevalence in younger people,^4^ compounds the need to identify biomarkers and methods to detect them, providing a diagnostic pathway that may be applied prior to the onset of motor symptoms.

Increased oiliness and flaky skin, especially on the face and scalp, are common symptoms of PD, first noted by Krestin in 1927.^5−7^ The light yellow, oily substance present on all human skin is known as sebum, and increased sebum production is a hallmark of PD. The sebaceous glands produce sebum in the skin, which helps keep the skin and hair moisturized, and prevent sweat from evaporating, thus assisting with the body’s temperature regulation. Sebum is an underexplored biofluid, which is readily obtained from non-invasive skin swabs, which primarily consists of a mixture of triglycerides, cholesterol, free fatty acids, waxy esters, and squalene.^8,9^ We have previously shown that sebum contains volatile compounds, which could be used as biomarkers of PD,^10,11^ and that it can reveal mitochondrial dysregulation as PD progresses.^12^ Here, we set out to develop a method to analyze sebum in its native state to facilitate rapid assessment of the PD status. Paper spray ionization mass spectrometry (PS-MS), which allows the direct analysis of compounds from paper, has previously been demonstrated to detect small molecules (50–800 Da) from unprocessed biofluids such as blood, urine, and CSF,^13−16^ but not to date with sebum.

Combining ambient ionization with ion mobility mass spectrometry has merit as a method to “clean up crude samples” and provides reproducible ion selected drift time data in the place of a retention time for identification along with *m*/*z*.^17−20^ PS-MS has already been mooted as a diagnostic method with clear advantages for inexpensive sampling and rapid analysis. Here, we couple paper spray ionization with ion mobility mass spectrometry (PS-IM-MS) and demonstrate for the first time its application for diagnostic feature discovery workflows, which could lead to methods of clinical utility. PS-IM-MS allows quick sample analysis with minimal sample processing compared to LC–MS, which here reveals far larger lipid moieties and provides enhanced separation for analytes with overlapping *m*/*z* ratios.

## Results and Discussion

### Developing an Optimized Workflow for Sebum Analysis

**Figure 1:**
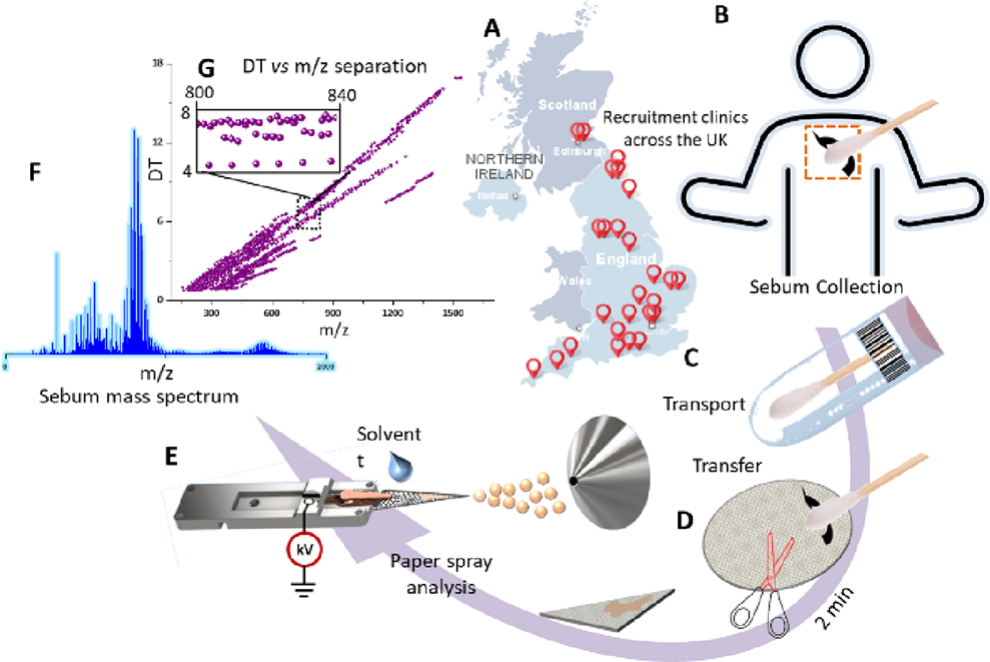
Workflow for paper spray analysis from clinic to raw data: (A) locations of collecting clinics in the United Kingdom, (B) sebum collection from the mid-back of participants, (C) medical Q-tips containing sebum samples transported under ambient conditions, (D) schematic of the sample transfer from the Q-tip onto the paper, (E) the paper spray process, (F) representative mass spectrum collected from sebum with a distinctive multimodal distribution, and (G) drift time vs *m*/*z* distribution with the zoom region exemplifying multiple drift time peaks associated with a specific *m*/*z* value.

The procedure developed for sample collection from sites across the United Kingdom and subsequent PS-IM-MS analysis is shown in Figure 1 (see Table S1 for further details on the sites).

**Figure 2:**
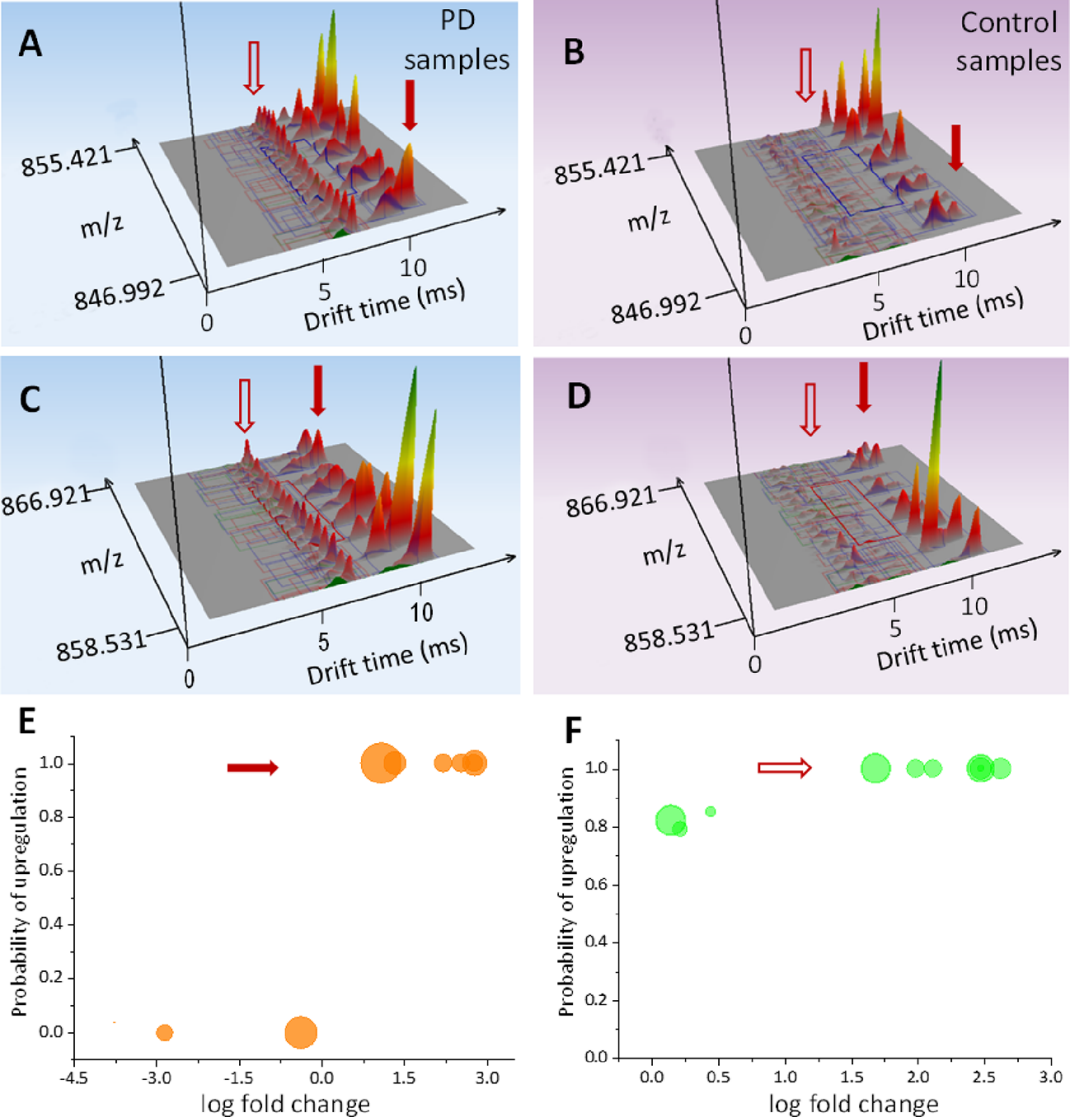
Three-dimensional DT vs *m*/*z* and intensity distributions for PD, *n* = 79, (A, C) and control, *n* = 71, (B, D) samples from ∼8 Da *m*/*z* windows exemplify the difference in the molecular composition of sebum produced by people with PD. Panels (A–D) report identical *m*/*z* and DT ranges with clusters of DT and *m*/*z* separated species associated with putative lipid features: 2.5–3.5 ms (Cluster 1), 3.5–5 ms (Cluster 2), 6–7 ms (Cluster 3), 7.5–9 ms (Cluster 4), and 9–10 ms (Cluster 5). Panels (A–D) show normalized averages of samples from PD (*n* = 79) and control (*n* = 71) participants from six recruiting sites. The red arrows indicate the clusters (3, open and 5, closed) that alter substantially between PD and control (Tables S3 and S4). Panels (E, F) show probability of up-regulation vs log-ratio for Cluster 5 (orange) and Cluster 3 (green); the area of the circles is proportional to 1/confidence interval.

Critical parameters of the PS-IM-MS experiments were optimized, which include the type, size, and shape of the paper, distance from the MS inlet, and eluting solvent composition (see the Materials and Methods section for further details). Crucial to reproducibility was how the sebum is transferred from the sampled Q-tip swab to the analytical/filter paper. Two methods were tested, first, by directly transferring from the Q-tip to the paper triangle in a “touch and roll” approach. Alternatively, we deployed rapid solvent extraction using the optimized PSI eluting solvent (ethanol, 800 μL). This involved vortex-mixing of the sampled Q-tip in ethanol for 5 s followed by spotting on to paper for analysis. Figure S1 shows mass spectra collected using these two approaches. Both are rich in features, and there is a notable increase in transmission of high molecular weight molecules (between *m*/*z* 1200 and 2000) in the touch and roll transfer mass spectrum (Figure S1A) versus the solvent extraction method (Figure S1B). Hence, this was chosen for all further sebum analyses (see the Materials and Methods section). Different eluting solvent mixtures were tested and optimal for sebum found to be 4.5 μL of H_2_O:EtOH (*v*:*v*, 4:1). Following these refinements, the mass spectra of human sebum consistently showed the presence of four distinct envelopes of predominantly singly charged species in the higher mass region (*m*/*z* 700–1800), with evidence of doubly charged species from isotopic distributions in highly congested feature-rich mass spectra (Figures 1F and 2A).

### Drift Time Separation Elucidates PD from Control

Ion mobility coupled to mass spectrometry is used here to resolve the high molecular weight species. Its application to resolve (and identify via collision cross section (CCS) matching) conformational isomers and isobaric structural isomers has been previously reported, albeit typically for lower molecular weight lipids.^21^Figure 1G shows a drift time (DT) vs *m*/*z* distribution of the ions generated and selected via Progenesis QI (Waters Corporation, UK), using default peak picking parameters, from a sebum sample of a PD patient. The richness of information from the IM-MS datasets is illustrated in Figure 1G (inset), in which 41 *m*/*z*-DT (4–8 ms) resolved ions can be detected within a 40 Da window (*m*/*z* 800–840). A mixture of features with very similar and identical *m*/*z* values is present within this range. In this work, we have utilized the separation power of ion mobility coupled with mass spectrometry for direct infusion to resolve high molecular weight features. In MS mode, prior to deconvolution, we typically identify 15 K ions from a single sample, and when we run in IM-MS mode, we detect just under 40 k. This 2.6-fold increase in the number of features found is critical to the discrimination that we get with the combined technique. By analyzing data from the full datasets from 150 patient and control samples, we observe ∼4200 *m*/*z*-DT features where 500 have a statistically significant relative abundance difference between PD and control samples (*p*-value < 0.05). Figure S2 displays the extracted arrival time distributions (ATDs) for ions measured at *m*/*z* 689.1 and 1394.8, showing the additional species resolved by IM. Enhanced separation between DT features for higher *m*/*z* values was achieved using a higher resolution SELECT SERIES cyclic IMS geometry (Waters Corporation) (data shown for *m*/*z* = 1394.8 (Figure S2B,C). A subset of the statistically significant molecules has drift time resolved features that are only observed in PD samples, and we, therefore, focused on their elucidation.

Figure 2A–D shows three-dimensional DT vs *m*/*z* distributions from *m*/*z* 700 to 900 for PD (blue) and control (magenta) samples. Five dominant regions of *m*/*z* and drift time aligned species with respect to drift/IM separation can be observed: 2.5–3.5 ms (Cluster 1), 3.5–5 ms (Cluster 2), 6–7 ms (Cluster 3), 7.5–9 ms (Cluster 4), and 9–10 ms (Cluster 5). Within these regions, species can be detected that differ in relative abundance between PD and control samples. These rich datasets require substantial interpretation as demonstrated below. Based on the isotopic spacing between the *m*/*z* detected species, we assign Cluster 1 as triply charged, Clusters 2 and 3 as doubly charged, and Clusters 4 and 5 as singly charged. These assignments are further supported when we convert DT to CCS values (Table S2) since the earlier arriving clusters (1–3) would result in CCS values that are too small for this particular *m*/*z* range if they would have been singly charged (see below). Within the *m*/*z* spectrum, there are repeating units in Cluster 3 that are separated by *m*/*z* 7.01, and within Clusters 4 and 5, the equivalent spacing is *m*/*z* 14.02, as revealed by inspection of drift time selected mass spectra (Figure S3A,B). These data taken together support assignment to a class of lipids that contains repeating units of CH_2_. Further to this, the mass spectrum shows that, for every lipid, there are at least four ions that differ by two mass units, which is due to different degrees of saturation within the hydrocarbon chains (Figure S4). Moreover, given that two singly charged clusters (4 and 5) can be detected, afforded by the IM-MS shape differentiation, suggests possible conformational differences for a number of the detected species. Tandem mass spectrometry experiments can aid to distinguish the isotopic and saturation features (Figure S5).

Significantly, Cluster 3 (hollow red arrow; Figure 2A,D) contains species that are abundant in samples from PD and not detected in controls (Figure 2A,C vs Figure 2B,D). Clusters 4 and 5 also show substantial differences in relative intensity between PD and control samples (discussed in detail later). These features resolved by DT that distinguish between PD and control samples are observed throughout ions detected in the *m*/*z* range of 700–900 (Figure S6). To confirm the results from Progenesis QI analysis, we also used an independent peak detection algorithm (Apex3D) and Bayesian statistics for complementary quantitative analysis. Figure 2E and Figure 2F show the probability of up-regulation vs log-ratio distribution for Clusters 5 and 3, respectively. The results are summarized in Tables S3 and S4, including the output from a Bayesian analysis^22^ of the components detected within the separate clusters (log fold change, confidence interval, and probability of up-regulation) as well as the outcome (*p*-value) of two-tailed *t*-test analyses of the same peak detected PS-IM-MS data. Table S3 reports the quantitative analysis of the components within Clusters 4 and 5, and Table S4 lists that for Cluster 3. This data shows that there is statistically significant up-regulation of these DT features in samples from PD patients.

### Annotation of Diagnostic Features from IM-MS–MS Datasets

To annotate these statistically important features in the DT vs *m*/*z* spectra, we employed accurate mass searching of available databases (HMDB and LipidMaps).^23,24^ These resources do not include experimental validation for lipids above a mass of 1400 Da and provide only scant information for those above 700 Da. Focusing first on the singly charged ions in the range of *m*/*z* 700–950 (Clusters 4 and 5) enabled tentative identification of multiple classes of lipids (Dataset S1) with the highest confidence assignment as triacylglycerides, C_*n*_H_*m*_O_*p*_, where *n* = 45–57, *m* = 84–104, and *p* = 6 over a mass range of 700–950 Da.

The region from *m*/*z* 500 to 650 is also rich in features, but fewer of these are statistically different in relative abundance between PD and control. We have found three regions (*m*/*z* 569.52, 597.55, and 611.57), where there are statistical differences and where the DT vs *m*/*z* distributions are also different by manual interpretation (Figure S7). Accurate mass searching of this region indicates that the lipid class most represented is diglycerides (DG), again with evidence of saturation in the hydrocarbon tails.

**Figure 3:**
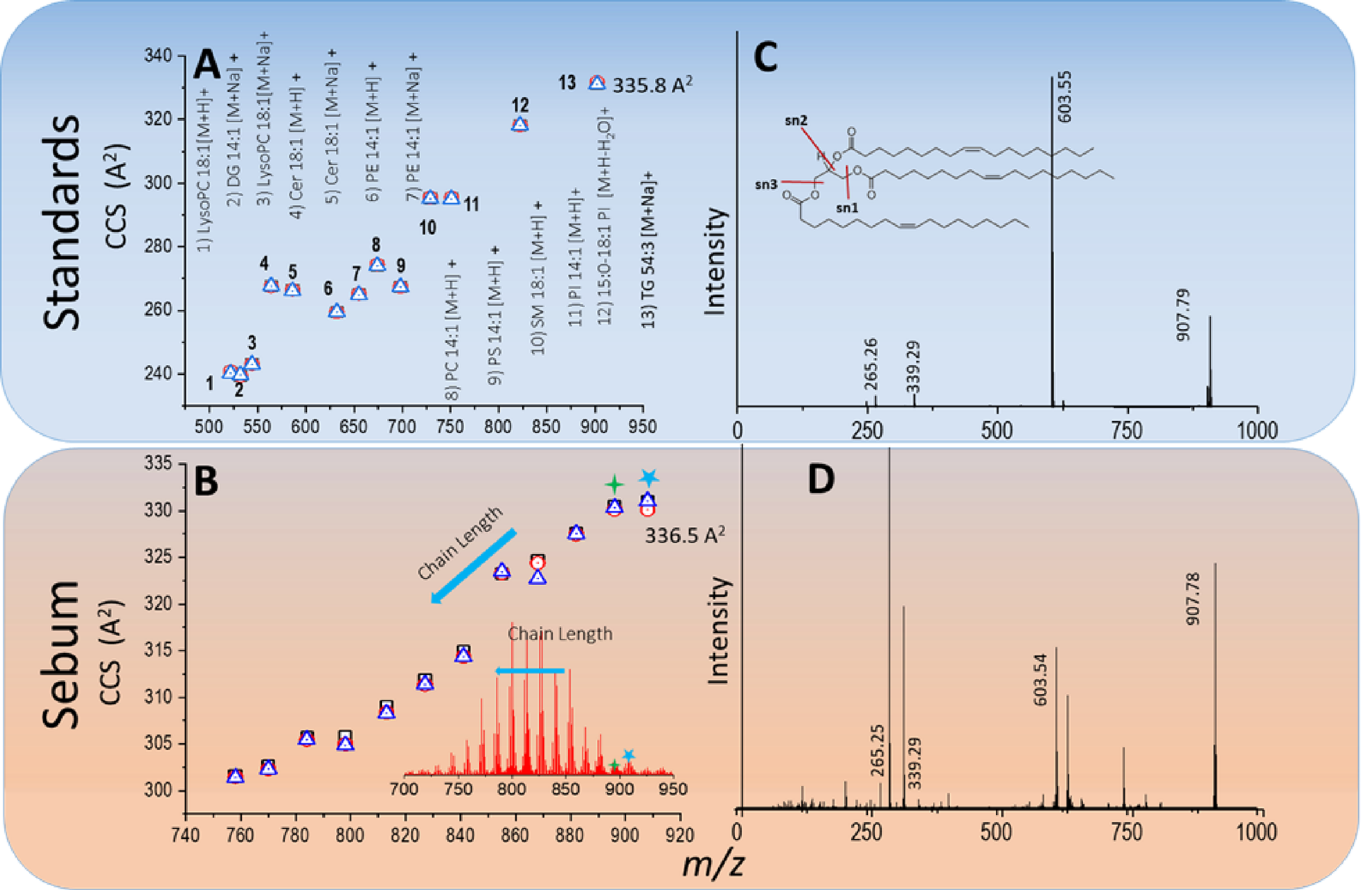
(A) CCS vs *m*/*z* distribution for a mixture of lipid standard 500–900 Da mass range. Data points numbered 1–13 show CCS values for different classes of lipids. Triplicate measurements were acquired under identical conditions (triangles, squares, and diamonds represent each measurement). (B) CCS vs *m*/*z* distribution for sebum. The inset shows the corresponding mass spectrum (*m*/*z* 700–950). (C) MSMS spectrum of triolein (TG 54:3), which is one of the components in the standard lipid mixture. The predominant fragments are labeled in the figure, corresponding to *m*/*z* 603.55, 339.29, and 265.26. (D) MSMS spectrum of a feature at *m*/*z* 907.78 in sebum (*n* = 1) showing highly similar fragmentation compared to that observed for the TG 54:3 lipid standard (C). All data presented in this figure were collected using PS-IM-MS using a higher resolution Cyclic IM-MS traveling wave instrument (Waters Corporation, UK).

Further annotation of these data used tandem MS as well as information from the ion mobility separation, *i.e.*, CCS, of the detected species. A commercially available lipid standard mixture was examined with PS-IM-MS using the same method. CCS values were obtained for the lipid species in this mixture with reference to the Major Mix calibrant (Waters Corporation, UK), and tandem mass spectra were recorded for the lipid standards. Both datasets were used to support identification of lipid classes in the sebum data. Figure 3A shows a CCS vs *m*/*z* distribution for species measured from the standard mix (see Figure S8 for the full mass spectrum). CCS values for adducted ions are reported for best comparison with the sebum measurements (Table S2). Figure 3B shows the CCS values obtained from the sebum lipids together with those from the standard mix.

Two lipids from the standard mixture fall within the mass region of interest in the sebum data, namely, those from the triacylglycerol (TG) and phosphatidylinositol (PI) lipid classes. The measured CCS values for the TG 54:3 [M + Na]^+^ (*m*/*z* 907.79) and PI 33:1 [M + H-H_2_O]^+^ (*m*/*z* 822.62) lipid components present in the standard mixture were 335.8 and 318.2 Å^2^, respectively (numbers 12 and 13 in Figure 3A). One of the features that falls within the higher mass region of interest in the sebum data is a species at *m*/*z* 907.79 (marked with an asterisk (*) in Figure 3B). The *m*/*z* and CCS values (336.5 Å^2^) of this sebum feature match those from TG 54:3 to <5 ppm and 0.2% CCS, respectively.

McLean *et al.* have previously reported CCS values for major lipid classes and demonstrate that the correlation of CCS with mass (as the chain lengths increase) differs due to head group chemistry and within each class on saturation of the hydrocarbon tails.^21^ A linear correlation in CCS values was observed in the sebum data for the series of peaks at 14 Da intervals (inset of Figure 3B), with a small decrease in CCS value going down the series. For example, the peak at *m*/*z* 895.77 (marked with + in Figure 3B) has a CCS value of 335.5 Å^2^. Hence, there is evidence that the ion mobility data presented in Figure 3B correlates to a mixture of TG lipids with varying fatty acid chain lengths. It is also known from the literature that the most significant components of human sebum are TG and fatty acids, comprising approximately 57.5%.^9,25^ Additionally, accurate mass and CCS matching were performed for the DG class of lipid features. For example, the accurate mass search of *m*/*z* 527.47 (one of the features seen in the *m*/*z* 500–650 region) suggests that it to be either DG (12:0/17:0/0:0) or DG (13:0/16:0/0:0) (mass error of 9.6 ppm in both cases). The measured CCS value for this species (249.2 Å^2^) is most comparable to the CCS value (243.3 Å^2^) of DG (14:1/14:1/0:0), present in the standard mixture (2.4% difference). Figure S9 shows the CCS vs *m*/*z* distribution in the region of *m*/*z* 500–650. In this case, a linear correlation was also observed for increases in the chain length of the attached hydrocarbons.

To increase confidence in our assignment and complement accurate mass and CCS measurements, tandem MS was utilized. Figure 3C shows the MSMS spectrum of *m*/*z* 907.79 (TG 54:3 [M + Na]^+^) measured from the mixture of standard lipids. Three predominant fragment peaks were measured at *m*/*z* 603.55, 339.29, and 265.26 corresponding to fragmentation in sn1, sn2, and sn3 positions, respectively. The sodium adduct of the TG 54:3 species is also present in sebum and was also selected for collisional activation (Figure 3D). The MSMS spectrum shows a high similarity in fragmentation to that obtained from the TG 54:3 standard (Figure 3C). The data from sebum is more complex than that obtained from the standard; this is because the precursor ions selected by mass are not chemically distinct, and there are isobaric species giving rise to some additional fragments. This complexity is evident from our IM data (Figure 2A–D) and from the complex fragment signature we obtain, which indicates differing chain lengths in these endogenous acyl glycerides. In our Cyclic IM-MS instrument, we are able to perform collisional activation post mobility separation, and this allows us to confirm the identity of a given precursor by comparing the ATD of fragment ions since they still possess the drift time of the precursor (Figure S10A). The ATDs of the TG signature fragment peaks (*m*/*z* 603.55, 339.29, and 265.26) are identical and are also identical to that of the precursor. A similar fragmentation behavior was found following tandem MS of other ions from sebum (Figure S10B), which yields the same general fragment ion series as the TG standard, albeit with some variation in the masses of the products by 14 Da units. In the standard we use, each of the three chains contains 18 carbons. The sebum data indicates that these lipids have variation in their acyl chain lengths.

The combination of three independent parameters, accurate mass, fragmentation data, and CCS value of the *m*/*z* 907.78 feature compared to the TG 54:3 standard enables lipid annotation at MSI level 1 (labeled with an asterisk (*) in Figure 3B).^26^ This annotation to the TG class can be applied to every other significant singly charged ion in the series between 700 and 950 *m*/*z* that supports their assignment as TG lipids with different fatty acid chain lengths and saturation. Linear correlation with incremental changes in CCS values also supports this conclusion. The IM-MS data indicates the presence of various extents of unsaturation in the fatty acid chains (discussed in detail below). As a negative control, we demonstrate the elimination of an alternative lipid class annotation of this series of peaks present/measured in sebum in the *m*/*z* region of 700–950 using MSMS data. A species at *m*/*z* 822.61 was detected in sebum, which matches closely in mass to PI 33:1, a component of the standard mixture (*m*/*z* 822.62, 11 ppm). The MSMS spectra of the standard PI 33:1 (Figure S11) show a loss of 259 Da, which is characteristic for this class of lipid via loss of the lipid head group. Fragmentation data from sebum for this species does not contain this loss and rules out PI lipid identification.

We have also observed the presence of diverse unsaturation in sebum lipids. Figure S12 shows a CCS value vs *m*/*z* distribution for the lipids present in the mass range of 700–900 Da. The small variation in CCS values of features very close in mass (circled with a dotted line) reflects changes in the number of double bonds present in the fatty acid chains as previously reported.^21^

### Putative Assignment of Higher Mass Features

One of the remarkable aspects of the PS-IM-MS data is the high molecular weight species, both Cluster 3 and the singly charged ions that occur in distributions that center on *m*/*z* 1080, 1334, and 1600. The relative intensity of these features does not change following dilution, ruling out aggregates formed during the ionization process. Focusing first on the significant doubly charged peaks that constitute Cluster 3 (Figure 2F, mass spectrum shown in Figure S3B), it is evident from the accurate masses and ATD data that these are not doubly charged dimeric clusters of the TG class of lipids that constitute Clusters 4 and 5. To verify this, the Cluster 3 *m*/*z* values were considered as solutions to the generalized form [M + A + B]^2+^, where A and B were variable adducts including H, Na, K, etc. A corresponding singly charged candidate peak list was generated of the form [M + A-H]^+^, [M + A-Na]^+^, [M + A-K]^+^, etc., which was queried against the peak detected data but did not match to singly charged ions in the mass spectrum. Searching COMP_DB of LipidMaps for these exact masses provides the best match identifications in the TG class, although these would require each of the three hydrocarbon tails to possess more than 30 carbon atoms on average.

Now, considering the high mass singly charged ions, the envelopes at *m*/*z* 1080, 1334, and 1600 are separated by 256 Da that can be evaluated as C_16_H_32_O_2_ (palmitic acid), which is also the separation between the *m*/*z* 850 region (TG) and the *m*/*z* 600 region (DG). This suggests that the sebum spectrum also contains tetra-, penta-, and hexa-acyl glycerides connected by ester linking additional C_12-18_H_24-32_O_2_ moieties. Such compounds have previously been identified in plants^27^ but not to date in humans.

Further experiments were used to elucidate these singly charged features observed in the *m*/*z* 1100–1700 region, including tandem MS post-IM. In such experiments, the ATD of the precursor ion is identical to that of its fragment. When performed on the entire spectrum, the relationship between the envelopes of features can be seen in breakdown curves (Figure S13), which predominantly dissociate via sn1, sn2, and sn3 cleavages into the lower order acyl glycerides. It is also possible to mass isolate a small group of *m*/*z* ions in the quadrupole prior to IM separation followed by tandem MS (Figure S14). By doing this, we observe that the large ions are composed of isomers of the general form *n*(C_12-18_H_24-32_O_2_) with different chain lengths and of course saturations.

The envelope of peaks in sebum differing by 14 Da has been assigned as a recurrent lipid series each changing by a single −CH_2_ unit. This would implicate the presence of lipids with odd number fatty acid carbon chains, which are uncommon in healthy serum but have been previously reported to be present in sebum,^25^ as well as more recently cited as indicators of a disease state.^28^ The origin of odd chained fatty acid (OCFA) such as C15:0 and C17:0 has been attributed to diet.^29^ It was recently suggested that OCFA ends up in propionyl CoA via mitochondrial β-oxidation of dietary OCFA. In contrast, even chains end up in acetyl CoA, having more physiological significance.^30^

In bloom-forming alga *Emiliania huxleyi*, unsupervised spatially aware clustering indicated a systemic metabolic shift toward lipids containing OCFA induced during viral infection.^31^ Similarly, a change in lipid metabolism is associated with disease phenotypes observed in a myriad of metabolomics studies from both plants and humans. Jenkins *et al.* suggested an endogenous biosynthetic pathway in human plasma that results in the ratio of pentadecanoic acid (C15:0) to heptadecanoic acid (C17:0) as 1:2 instead of the expected 2:1 as seen in dairy fat.^30^ Venn-Watson *et al*. stated that C15:0 may not be endogenously produced as readily as C17:0 and is more likely obtained from dairy, fish, and plant-rich diets,^29^ where lower intake of C15:0 is associated with higher mortality. The study postulates that C15:0 is an essential fatty acid with a metabolic role in repairing mitochondrial function to alleviate inflammation, anemia, dyslipidaemia, and fibrosis *in vivo*. Other investigations have suggested that the presence of OCFA in serum is associated with reduced risks of metabolic diseases,^32,33^ and recent work using imaging mass spectrometry and ion mobility mass spectrometry indicated that OFCA is strongly associated with cancer cell growth.^28^

The role of lipids in PD pathogenesis has been implicated in several studies. Increased levels of PA (18:2/15:0) in plasma have been cited as potential markers of PD.^34^ PA selectively binds to α-synuclein and has been shown to induce its aggregation,^35^ and skin punch biopsies have indicated higher levels of α-synuclein in People with Parkinson’s (PwP),^36,37^ which hints at a role for the altered lipid content we observe in the skin brain etiology of this disease. The gut microbiome of PwP has a lower amount of butyrate-producing bacteria as well as lower concentrations of fecal short-chain fatty acids (SCFA), which is hypothesized to influence the gut-brain axis.^38,39^ While these and other studies have highlighted the role of short-chain fatty acids in the manifestation of PD,^38,40^ very little has been reported regarding OCFA. A recent meta-analysis revealed lower amounts of triglycerides in the serum of male patients with Parkinson’s in contrast to our findings, which may be due to the differences in the fluid sampled (serum vs sebum) and/or in the analytical method used.^41^ All eight studies in the meta-analysis used lipase assays that assess the total TG content rather than MS metabolomics, which has far better selectivity for given analytes, and in our analyses of sebum, only ca. 10% of the features measured are different between PD and control. We have previously shown that the dysregulations of lipids and mitochondrial function are critical perturbations observed in sebum from PD participants.^12^ The new results presented here indicate that sebum, which acts to remove excessive metabolites from the lymphatic system, may well be the key to understanding the changes in regulation of these essential fatty acids as PD progresses.

## Conclusions

In conclusion, a new and accessible method to analyze sebum samples has been developed and shown to be able to readily distinguish between samples taken from PwP versus matched controls. PS-IM-MS of each sebum sample is performed in ∼2–3 min, which is noticeably faster than current clinical mass spectrometry approaches. Previous studies have demonstrated the use of PS-MS to detect metabolites present in the blood, urine, and other biofluids,^14−16^ and it has not previously been applied to sebum. These results, coupled with the lower signal from high mass species following solvent extraction for classic xC-MS studies, also demonstrate the benefit of analysis with ambient ionization direct from the native biofluid. Liquid chromatography-mass spectrometry (LC–MS) was not able to detect such large molecular weight species (>1200 Da) from sebum extracts.^12^

Mass spectra of sebum samples acquired using PS-MS demonstrated the technique’s utility measuring both low and high molecular weight species (*m*/*z* 50–2000) that may be lost in sample preparation, namely, solvent extraction and/or stick to LC columns, in more traditional analytical methods. PS-MS combined with IM separation reveals specific compounds unique to PD sebum samples when compared to healthy controls. Furthermore, we have identified two classes of lipids, namely, triacylglycerides and diglycerides, as components of human sebum that are significantly differentially expressed in PD. Non-invasive sampling followed by PS-IM-MS analysis targeting these compounds could provide an inexpensive assay to support clinical phenotyping for the confirmatory diagnosis of Parkinson’s disease.

## Materials and Methods

For paper spray measurements, grade 1 and 42 filter papers were used (Whatman International Ltd., UK). The paper was cut into isosceles triangles at 5 mm (base) × 10 mm (height). LC–MS grade solvents were used for the study. This included acetonitrile (98% purity), methanol (99% purity), deionized water (Fisher Scientific, UK), and ethanol (99% purity) (VWR Chemicals, UK). Solvents were used without any further purification. An ESI-L low concentration tuning mix (TM) (Agilent Technologies, UK), l-glutamine, and l-proline (Sigma-Aldrich, UK) were used as standards for optimization of the process. For tandem mass spectrometric experiments, a range of commercially available standard mixtures of different lipid classes (Differential Ion Mobility System Suitability Synthetic Standard Mixture and LightSPLASH LIPIDOMIX Quantitative Mass Spec Primary Standard (Avanti Polar Lipids, Inc., USA) were purchased. For direct infusion mass spectrometry, the salt content is an important factor as the presence of excess salt in samples is not favorable for better ionization. Hence, desalination (using Ziptips C18) and dilution of the standards were performed prior to PSI-IM-MS measurement using a traveling wave instrument (Synapt G2-Si and SELECT SERIES cyclic IMS, Waters Corporation, UK). The instruments were mass- and CCS-calibrated prior to the sample measurements. CCS data and tandem mass (MSMS) spectra were recorded for the lipid standard infused via PS-MS.

A source was designed in-house (using Autodesk Inventor 2018) and 3D printed (Ultimaker 3 Extended, GoPrint3D, Ripon, UK) for paper spray analysis on a Synapt G2-Si instrument. Copper micro-alligator clips (Premier Farnell UK Ltd., UK) were used to hold the paper triangles at a high potential followed by positioning them close to the MS inlet. Medical Q-tips swabs (Fisher Scientific, UK and Copan Diagnostics, USA) were used for sample collection.

### Study Participants

For the initial method development of PS-MS using sebum, samples from healthy controls were used. The developed method was then applied to samples from participants with PD and a similar number of control participants. The participants for this study were part of a recruitment process at 27 NHS clinics all over the United Kingdom (Table S1). A total of 150 participant samples were used in this investigation, and the statistical analysis of the data is based on this entire set. An overview of important patient demographics is summarized in Table S5. Of our 150 participants, 64 were from a single site (34 PD, 30 control) and a further 86 samples from five different collection sites. We were hence able to evaluate any site or collection bias (see below). The results of significance tests between cohort group metadata are reported in Table S6 . Ethical approval for this project (IRAS project ID 191917) was obtained from the NHS Health Research Authority (REC reference: 15/SW/0354).

### Sample Collection

Sebum samples were non-invasively swabbed from the mid-back of participants with medical Q-tip swabs. Each swab, secured in its holder, was transported under ambient conditions in sealed envelopes to the central facility at the University of Manchester, where they were stored at −80 °C until analysis.

### Paper Spray Ionization Mass Spectrometry (PS-MS)

The size and shape of the paper triangles were found to be critical to achieving reproducible data, indicating that the biomass of the sampling surface and the applied sebum can be controlled. Paper triangles were cut manually, and each one was quality-controlled for size prior to use. A camera attached to the source housing ensured reproducible positioning of the paper tip for each measurement. An optimized position was marked, and the paper tip was placed at that mark for each repeat. Whatman grade 1 and 42 filter papers were tested for PSI-MS experiments using standards (TM, l-proline, and l-glutamine). Following optimization, the total ion chromatogram (TIC) and the mass spectra acquired using each filter paper were visually similar (Figure S15), although reproducibility was higher with the Whatman grade 42 paper. Relative standard deviation (RSD) in repeated measurements of the standards was 9.6 using Whatman grade 42 paper and 15.06 using Whatman grade 1 paper (Figure S16); Whatman grade 42 paper was used for all further analysis. Reproducibility of PS-MS for sebum was also tested over multiple runs throughout a day under identical instrumental conditions. Figure S17 represents mass spectra recorded for sebum at 8 h intervals. No significant shift in the masses was observed.

Sebum was transferred from the Q-tip swabs onto the paper substrates by gentle touch and roll of the swab onto the sampling area. After sample transfer, the paper triangle was clipped onto the copper alligator clip using tweezers avoiding contamination. Each copper clip was cleaned by ultrasonication in acetone before use. For each sample, a new clip and tweezer were employed to prevent cross-contamination. The clip was connected to a custom paper spray ion source built in-house and adapted to a Synapt G2 Si HDMS ion mobility mass spectrometer. PS-MS measurements were commenced by positioning the paper tip in front of the MS inlet using a movable *xyz* nESI stage and subsequently applying a voltage (2.5–3 kV) to the alligator clip by adapting the ESI capillary voltage supply. Upon elution with a polar solvent at that elevated potential, a spray plume of tiny charged droplets was observable at the tip of the paper simultaneously with the appearance of an ion signal.

All mass spectra were recorded over the range of *m*/*z* 50–2000. The critical instrument parameters for each PS-IM-MS experiment were capillary voltage at 2.5 kV, source temperature at 80 °C, sampling cone at 30 V, source offset of 40 V, IMS wave velocity of 650 m/s, and IMS wave height of 40 V. No desolvation or cone gas was used. Mass spectra were recorded for 2 min at a scan rate of 2 s per scan. A total of 60 scans were used for further data analysis.

Sebum was measured on the same day as the standard mixture under the same experimental conditions and using the same CCS calibration to target statistically significant features for CCS value calculation.

Tandem MS was performed using the Cyclic Select Ion Series MS with nitrogen as the collision gas. In all measurements, the collision energy (CE) was manually adjusted to determine optimal fragmentation conditions for each lipid class (Figure S13). This information was utilized in further comparative studies where the CE was held constant at the appropriate voltage, as reported in Figure 3 and Figures S10 and S11.

### Use of Internal Standards

To check the reproducibility of paper spray across different samples, TM was used. For these experiments, 3.5 μL of the TM solution was spotted on paper triangles and air-dried. Dried paper triangles were used for PSI-MS measurements of sebum samples following an identical method described in the previous paragraph.

### Limit of Detection

The amount of biomass present on the Q-tip depends on the following facts. First, the production of sebum between people can be variable, people with PD often suffer from Seborrhea, which affects ca. 5% of the adult population, and sebum production is higher in men than women and higher in the elderly. Therefore, if a sample is collected from a participant with low sebum production, then the biomass on the Q-tip will be low. Second, sample collection efficiency also varies from person to person. A limit of detection experiment was performed to test our method for various amounts of biomass present on the Q-tip. In this experiment, an unknown quantity of sebum was extracted from a Q-tip into a known amount of solvent (using the solvent extraction method discussed earlier). This extracted stock was then repeatedly diluted by 50% until the features of sebum were not visible in the mass spectrum. A known internal standard (a standard lipid) was added for our reference. A relative concentration of sebum was calculated from the absolute intensities in the mass spectrum. In this experiment, we could detect as low as 500 pM sebum. Below this concentration, sebum features were not distinguishable from the noise in the mass spectrum. We have previously demonstrated with GC–MS that variances in sebum production between participants fall within a dynamic range where we have linearity of response for data acquisition. Following deconvolution and normalization, we see no bias in subsequent analysis.^11^

### Data Processing with Progenesis QI

After recording the IM-MS data from all the participant samples under identical conditions, the raw data were peak-detected and deconvolved using Progenesis QI (Nonlinear Dynamics, UK). Peak picking initially identifies accurate mass *m*/*z* values and coincident ion mobility drift times (DT). These correlated *m*/*z*-DT features are then aligned, and area normalization is carried out with reference to the best candidate sample, within the entire dataset, chosen by a default set of parameters. Peak picking limits were set to automatic with default noise levels to balance the signal-to-noise ratio according to the data quality. The signal acquired before 0.1 min of infusion and after 1.4 min of infusion was discarded during processing to only retain reproducible signals.

For initial quantitative analysis to determine regions of interest, Progenesis QI was applied, which nominates one of the PS-MS raw data files a reference to which the other runs are normalized by determining so-called individual, run-specific scalar factors. Typically, the data file with the largest number of features is chosen as the reference. To support our assumption that our analytical approach is not biased by differences in biomass on sebum sampling nor between PD and control cohorts, we plotted the summed intensities of the picked features (Figure S18). We see a variation in the TIC values found from each individual sampled that is irrespective of whether the sample came from a PD patient or a control subject. This indicates that the found features that discriminate PD from control are not dependent on the amount of material sampled.

### Feature Selection Using Progenesis and Apex3D

Using Progenesis, features were classified as significant if their *p*-value was less than 0.05, determined by one-way analysis of variance (ANOVA). To allow subsequent annotation of features of interest, accurate mass features were matched with both the Human Metabolome Database (HMDB) and LipidMaps.^21,22^ The use of fold changes and ANOVA testing was used along with visual identification of *m*/*z* and drift time resolved regions of interest that afford differentiation between sample groups. The information residing in the regions of interest was further analyzed using an established probabilistic (Bayesian) method^22^ that was specifically designed for data of the nature typically associated with direct infusion MS experiments. The choice of priors includes the reliability (p) of the assignment of the data to the analytes and the scale of the intensities (Λ) that are measured. The Markov chain Monte Carlo settings were Gibbs sampling with 100 iterations.^22^ This method can accommodate uncertainty in feature assignments and can quantify the uncertainty in the results. At this stage of the analysis of the data, a molecular feature is a mass, drift time pair. To do this, IM-MS data were peak-detected using Apex3D, embedded in Progenesis QI for metabolomics (Nonlinear Dynamics, UK). The processed data were single-point *m*/*z* corrected using the singly charged monoisotopic mass of hexakis(2,2,3,3-tetrafluoropropoxy)phosphazene from Tune Mix (Agilent Technologies, CA) and total ion current (TIC) normalized. Quantitation of the *m*/*z* and drift-separated components of interest, with analyte CCS cluster indices and ranges listed in Tables S3 and S4, was conducted using a previously described probability-based framework.^22^ Measurement noise was assessed by nominating drift-separated low *m*/*z* PS-MS matrix ions (103.05 Th/1.0 ms, 105.07 Th/1.0 ms, and 109.10 Th/1.2 ms) as pseudo internal standards. Dataset S2 contains the data to show that the biomass of sebum does not correlate to PD or control and that the pseudo standard ions from each participant do not exhibit statistically significant changes in their relative intensities.

### CCS Calibration

The data were calibrated using a recently described approach, which gives improved performance for multiply charged analytes in TWIM devices.^42,43^ The reference peaks used to create the calibrations were selected from Tune Mix^44,45^ (Synapt G2-Si data), singly and doubly charged polyalanine,^46^ and MajorMix^47^ (SELECT SERIES cyclic IMS data). CCS reference peaks were identified by accurate mass in peak lists generated using the Apex3D algorithm, and the calibrations were created and applied using the IMSCal software.^43^ For both datasets, the velocity relaxation parameter “*a*” was fixed at 1.0, and for the Synapt G2-Si instrument data, the radial/RF parameter “*c*” was set at 0.2. Figure S19 shows the uncertainty distributions associated with the CCS calibration for the lipid clusters of interest.

### Investigation of Site and Sampling Effects

To determine how this approach could be widely adopted, we used the entire dataset of 150 participants to investigate the influence of location or the person who collected sebum on the data. Of these 150 participants, 64 were from a single site (34 PD, 30 control) and a further 86 samples from five different collection sites. Principal component analysis (PCA) (Figure S20), support vector machine (SVM), and random forest (RF) modeling classified samples according to collection sites. No apparent separation was possible using PCA, but this is often the case for complex data since PCA is an unsupervised dimension reduction method. To assess supervised learning, the samples were split 100 times into statistical training (75%) and test (25%) sets. For each split, the model under consideration was trained on the training set and then tested using the test set. The prediction output from each test was output as a confusion matrix. Finally, a confusion matrix representing the average of 100 tests was reported (Tables S7 and S8 for SVM and RF models, respectively). This analysis suggests that this data cannot be classified by the collection site; further, since samples from different sites and patients were acquired on different days, we surmise that PSI-IM-MS can be applied to detect differences in the molecular composition of sebum that can diagnose PD without influence from the sampling environment nor batching effects.

## Supporting Information Available

Dataset S1: The IM-MS datasets generated during and/or analyzed during the current study (XLSX)
Demographic details of the collecting sites; mass spectrometry, tandem mass spectrometry, and ion mobility mass spectrometry datasets from samples and from standards; statistical significance between PD and control cohorts; reproducibility tests; confusion matrix using an SVM and an RF model (PDF)
Dataset S2: Comparison of signals from PD and control and pseudo standard ions from each participant that do not exhibit statistically significant changes in their relative intensities (XLSX)

## ABBREVIATIONS

PD: Parkinson’s disease
PS-IM-MS: spray ionization coupled with ion mobility mass spectrometry
PS-MS: paper spray ionization mass spectrometry
CCS: collision cross section
DT: drift time
TG: triacylglycerol
PI: phosphatidylinositol
DG: diglyceride
ATD: arrival time distribution
OCFA: odd-chained fatty acid
PwP: People with Parkinson’s
SCFA: short-chain fatty acids
SVM: support vector machine
RF: random forest
