# Supplementary material for: Paper Spray Ionization Ion Mobility Mass Spectrometry of Sebum Classifies Biomarker Classes for the Diagnosis of Parkinson’s Disease
Source: JACS Au. 2022 Sep 7;2(9):2013–22. doi: 10.1021/jacsau.2c00300 (PMC9516698; doi:10.1021/jacsau.2c00300)
Supplement: Supplementary file 2 — au2c00300_si_002.pdf [file au2c00300_si_002.pdf]

Paper Spray Ionisation Ion Mobility Mass Spectrometry of Sebum Classifies Biomarker Classes for the Diagnosis of Parkinson's Disease

Depanjan Sarkar[1], Eleanor Sinclair[1], Sze Hway Lim[2], Caitlin Walton-Doyle[1], Kaneez Jafri[1], Joy Milne[1], Johannes P.C. Vissers[3], Keith Richardson[3], Drupad K. Trivedi[1], Monty Silverdale[2], and Perdita Barran[1]*

[1]Manchester Institute of Biotechnology, School of Chemistry, the University of Manchester, Princess Street, Manchester, UK, M1 7DN.

[2]Department of Neurology, Salford Royal Foundation Trust, Manchester Academic Health Science Centre, University of Manchester, UK, M13 9NQ.

[3]Waters Corporation, Stamford Avenue | Altrincham Road, Wilmslow, SK9 4AX UK.

AUTHOR INFORMATION

Corresponding Author

Perdita Barran

Manchester Institute of Biotechnology, School of Chemistry, the University of Manchester, Princess Street, Manchester, UK, M1 7DN.

Email: perdita.barran@manchester.ac.uk.

Table of Contents

Table 1 Demographic details of the collecting sites in the UK and the lead PI at each site. 4

Figure S1. Paper spray mass spectra collected from sebum using A) touch and roll transfer and B) quick extraction in 100% EtOH, indicating the presence of higher mass molecules (in between m/z 1200-2000) in case of touch and roll transfer. 5

Figure S2. Arrival time distributions for selected m/z values 689.1 (A) and 1394.8 (B) using linear TWIG IM-MS (Synapt G2-Si). C) Arrival time distribution for m/z 1394.1 using a cyclic TWIG IMS (SELECT SERIES cyclic IMS) instrument. 6

Table 2. IM-MS separated clusters and tentatively identified lipids and detected $^{TW}CCS_{N2}$ ranges...7

Figure S3. Drift time selected mass spectra from Clusters 4 and 5, with ions separated by 14.02 Da. (A) and from Cluster 3 where the separation is 7.01 Da. (B). This, as well as the 0.5 Da spacing within each isotopic distribution, supports the assignment of species in Cluster 3 as doubly charged 8

Figure S4. Experimental (A) and theoretical (B) example isotopic distribution within *m/z* 850-860 showing the presence of a different degree of unsaturation in lipids detected in sebum................8

Figure S5. Data from tandem mass spectrometry experiments performed on ions isolated following direct infusion ionisation of sebum showing that in each lipid case reported here there are at least 4 ions that differ by 2 mass units, due to different degrees of unsaturation within the hydrocarbon chains. A) Full MSMS spectrum of the selected ions showing all the fragments, and B) zoomed view of the selected *m/z* region of 700-900 displaying the change in isotope distribution. 9

Figure S6. Three-dimensional DT *vs. m/z* and intensity distributions for PD (*n*=79) (blue) and control (*n*=71) (magenta) samples from other regions in the DT M/z plots of statistical significance from m/z 840-880. The hollow arrow indicates the region, drift time ~6.7 ms, where there are a series of doubly charged ions that are only present with significant intensity in the PD samples. The two solid arrows indicate the later arriving singly charged species that also present with significantly different intensities between PD and control. 10

Table 3. Differential expression of conformation/configuration clusters of identified lipids by means of PS-IM-MS. 11

Table 4. Differential expression of doubly charged conformation/configuration (Cluster 3) of lipids detected by means of PS-IM-MS. 12

Figure S7. Three-dimensional DT *vs. m/z* and intensity distributions for PD (*n*=79) (blue) and control (*n*=71) (magenta) samples for regions of statistical significance from *m/z* 500-650. 13

Figure S8. A mass spectrum collected of the standard lipid mixture LightSPLASH™ LIPIDOMIX® Quantitative Mass Spec Primary Standard (Avanti Polar Lipids, Inc., USA), numbers refer to different classes of lipids present in the mix (please refer to Figure 3A in main text for the identities of the numbered ions). 14

Figure S9. Plots that shows $^{TW}CCS_{N2}$ values *vs. m/z* for ions found in sebum in the *m/z* region 500-650. 14

Figure S10. MSMS spectra from different precursor ions present in sebum, denoted by the mass values RHS occurring at 14 m/z intervals wrt x axis. The inset shows a zoomed view of the spectrum from *m/z* 100-400. Fragment ions at *m/z* 339.29, and 265.26 are common in all these spectra. This implies that two of the fatty acid chains in the TG class of lipid are common and the chain length varies on the third fatty acid. 16

Figure S11. MSMS of PI 33:1 showing a characteristic loss of 259 Da of the polar head group of the PI lipid class...17

Figure S12. CCS value *vs. m/z* for the lipids present in the mass range 700-900 Da. 17

Figure S13. Extracted ion breakdown curves for Penta-acyl glyceride, Tetra-acyl glyceride, Hexa-acyl glyceride, Diglyceride, and Triglyceride with increasing collision energy. The data for this TIC was normalised from data where the collision energy was ramped and then fitted to polynomial curves using Origin Lab21. 18

Figure S14. MSMS spectra of selected precursor ions from different regions in the sebum spectrum A) triglyceride, B) tetra-acyl glyceride, C) penta-acyl glyceride, and D) hexa-acyl glyceride. 19

Table 5. Demographics of participants included in data analysis..20

Table 6. Statistical significance between PD and control cohorts...21

Figure S15. A-B) Total ion chromatogram (TIC) of tune mix (Agilent) using PS-MS from Whatman 42 and Whatman 1 filter papers, respectively. C-D) Corresponding average mass spectra. The TIC and the mass spectra acquired using each filter paper were visually similar, although reproducibility was higher using the Whatman grade 42 paper. 22

Figure S16. Reproducibility test using a set of 10 samples (L-glutamine under identical conditions), from Whatman 1 and 42 filter papers. 22

Figure S17. PS-MS mass spectrum of sebum from the same individual recorded at bottom 0h and top 8h intervals. 23

Figure S18. Plot of summed intensities (64 samples) of the features picked by Progenesis QI. Black: PD samples, red: control samples, and blue: blank paper…………………………………………………………21

Figure S19. The CCS data were calibrated using a recently reported approach, which gives improved performance for multiply-charged analytes in TWIM devices.[1] CCS calibration uncertainty. Colour = cluster index (red = 1; blue = 2; green = 3; yellow = 4; orange = 5). 24

Figure S20. Principal component analysis for PD samples from 5 different recruitment sites (10 samples from each site). The lack of clustering indicates that there is no bias to a given site in the method. 25

Table 7. Confusion matrix using a support vector machine (SVM) model reporting the average prediction output of classifying samples based on collection site (n=100). 26

Table 8. Confusion matrix using a random forest (RF) model reporting the average prediction output of classifying samples based on collection site (*n*=100). 27

Centre ID	Centre Name	PI
**01**	Royal Bournemouth General Hospital	Mary Smolen
**02**	Southern Health Foundation Trust	Matthew Young
**03**	South Tees Hospitals NHS Foundation Trust	Sarah Morris
**04**	Salford Royal NHS Foundation Trust	Monty Silverdale
**05**	Nottingham University Hospitals	Gillain Sare
**06**	Western General, Edinburgh	Gordon Duncan
**07**	Hampshire Hospitals Foundation Trust	Deborah Dellafera
**08**	Cambridge University Hospital	Rachel Ahmed
**09**	Sheffield	Rosie Clegg
**10**	Bury	Judith Brooke
**11**	Royal Cornwall Hospitals	Ali James
**12**	Salisbury	Alpha Anthony
**13**	London	Cheryl Pavel
**14**	London	
**15**	Luton & Dunstable	Yvonne Croucher
**16**	Portsmouth	Catherine Edwards
**17**	Bath	Elizabeth Whelan
**18**	North Tyneside/Northumbria	Steve Dodds
**19**	MRC Centre for Regenerative Medicine, Edinburgh	Tilo Kunath
**20**	Seb Derm, Edinburgh	Richard Walker
**21**	Amsterdam, NL	Anouk Rijs
**22**	JDR, Manchester	Dani Mounfield
**23**	Plymouth	Catherine Pitman/Sandra Morgan
**24**	Sunderland	Anita Rutkauskaite
**25**	Devon	Rob James
**26**	Gateshead	Bryony Storey
**27**	Newcastle upon Tyne	Alison Sutherland
**28**	Imperial College	Ruby Colley

Table 1 Demographic details of the collecting sites in the UK and the lead PI at each site.

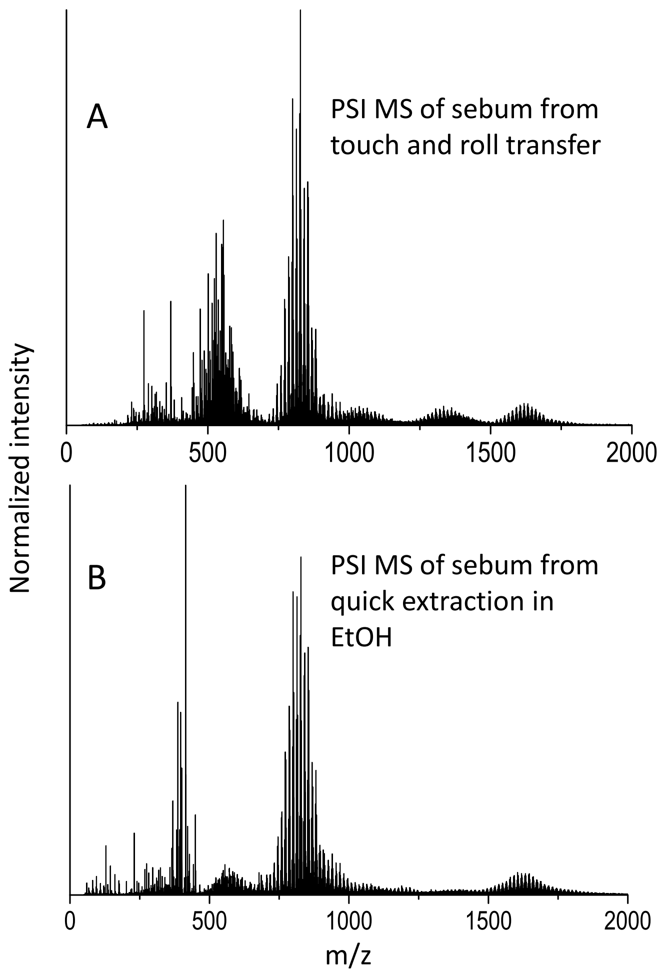

Figure S1. Paper spray mass spectra collected from sebum using A) touch and roll transfer and B) quick extraction in 100% EtOH, indicating the presence of higher mass molecules (in between m/z 1200-2000) in case of touch and roll transfer.

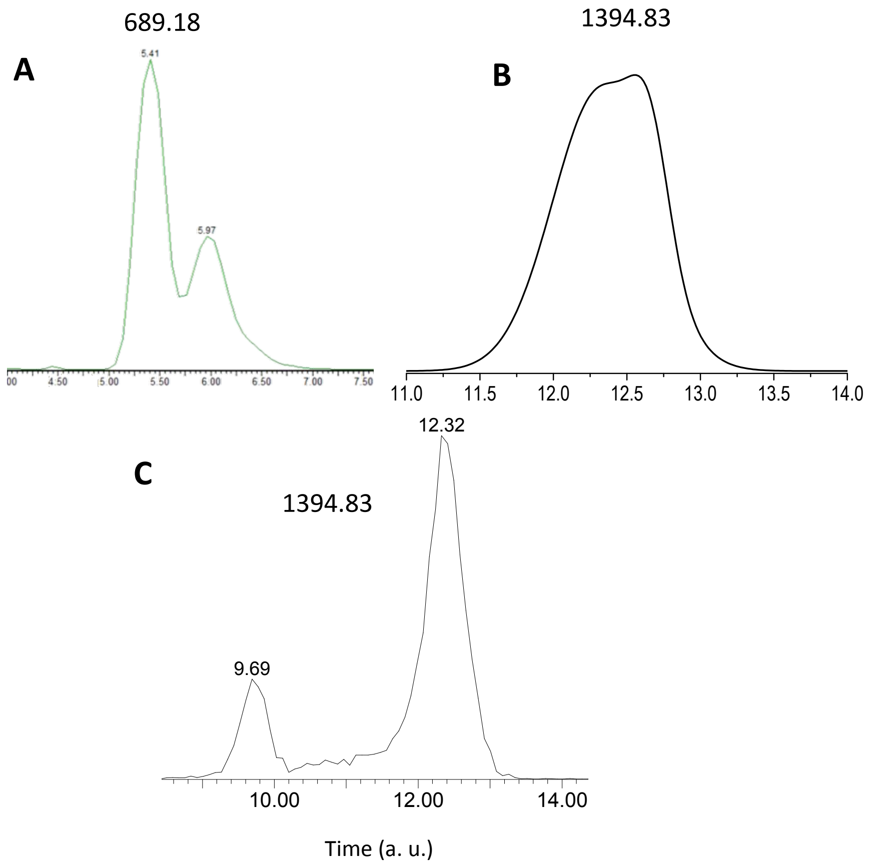

Figure S2. Arrival time distributions for selected m/z values 689.1 (A) and 1394.8 (B) using linear TWIG IM-MS (Synapt G2-Si). C) Arrival time distribution for m/z 1394.1 using a cyclic TWIG IMS (SELECT SERIES cyclic IMS) instrument.

			$^{TW}CCS_{N2}$ (Å^2) ranges singly charged conformer/configuration cluster(s)				
lipid*	adduct	calculated *m/z*†	1	2	3	4	5
TG 42:1	$[M + Na]^+$	720.6268	--	188.2 - 198.8	232.3 - 235.6	265.1 - 266.9	289.7 - 293.4
TG 43:1	$[M + Na]^+$	734.6424	166.7 - 166.7	189.8 - 202.8	234.1 - 235.8	267.7 - 267.7	293.7 - 296.1
TG 44:1	$[M + Na]^+$	748.6581	168.5 - 168.5	185.9 - 211.8	236.7 - 238.8	--	297.0 - 300.5
TG 45:1	$[M + Na]^+$	762.6737	169.1 - 169.2	190.2 - 199.2	238.7 - 241.6	275.4 - 275.4	300.2 - 303.6
TG 46:1	$[M + Na]^+$	776.6894	171.4 - 173.4	188.6 - 214.5	242.2 - 243.5	--	303.7 - 307.0
TG 47:1	$[M + Na]^+$	790.7050	163.6 - 180.2	195.5 - 217.2	244.9 - 245.4	277.4 - 277.4	306.6 - 310.2
TG 48:1	$[M + Na]^+$	804.7207	165.2 - 179.3	194.9 - 218.6	247.6 - 247.6	--	310.1 - 313.3
TG 49:1	$[M + Na]^+$	818.7363	168.2 - 175.8	193.9 - 226.5	--	285.7 - 285.7	315.4 - 315.4
TG 50:1	$[M + Na]^+$	832.7520	167.6 - 179.3	201.1 - 221.4	252.0 - 252.0	--	318.8 - 319.5
TG 51:1	$[M + Na]^+$	846.7676	177.8 - 180.2	211.2 - 226.7	--	--	--
TG 52:1	$[M + Na]^+$	860.7833	179.4 - 185.0	211.4 - 231.4	--	289.6 - 289.6	324.3 - 324.8
TG 53:1	$[M + Na]^+$	874.7989	185.0 - 191.5	208.9 - 226.8	--	291.4 - 291.4	--
TG 54:3	$[M + Na]^+$	884.7833	182.6 - 194.6	209.5 - 236.5	258.0 - 260.8	--	325.4 - 330.1

* tentative identification/annotation; † LipidMaps (https://www.lipidmaps.org/); -- = not detected

Table 2. IM-MS separated clusters and tentatively identified lipids and detected $^{TW}CCS_{N2}$ ranges

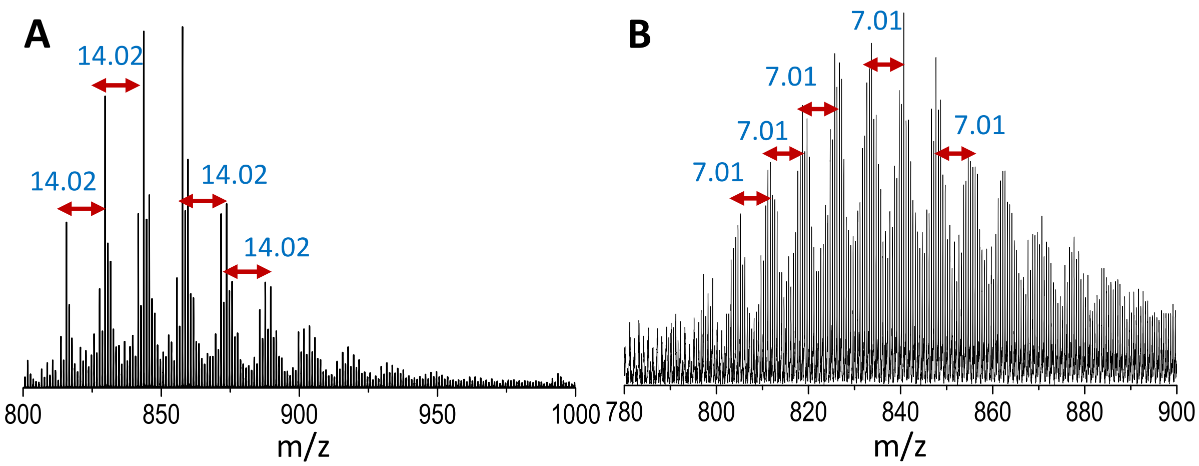

Figure S3. Drift time selected mass spectra from Clusters 4 and 5, with ions separated by 14.02 Da. (A) and from Cluster 3 where the separation is 7.01 Da. (B). This, as well as the 0.5 Da spacing within each isotopic distribution, supports the assignment of species in Cluster 3 as doubly charged.

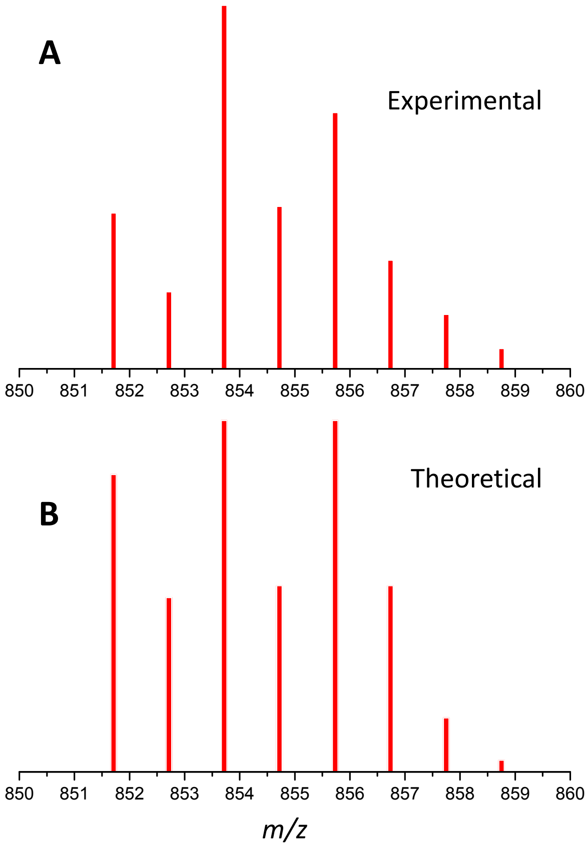

Figure S4. Experimental (A) and theoretical (B) example isotopic distribution within *m/z* 850-860 showing the presence of a different degree of unsaturation in lipids detected in sebum.

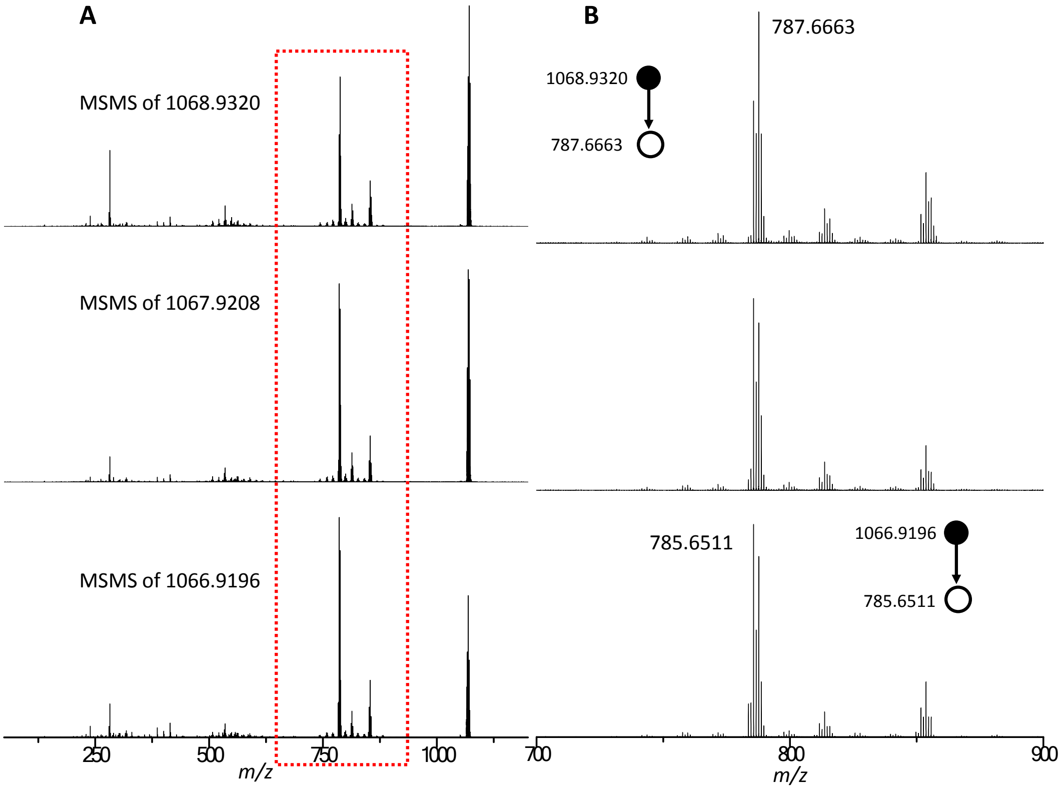

Figure S5. Data from tandem mass spectrometry experiments performed on ions isolated following direct infusion ionisation of sebum showing that in each lipid case reported here there are at least 4 ions that differ by 2 mass units, due to different degrees of unsaturation within the hydrocarbon chains. A) Full MSMS spectrum of the selected ions showing all the fragments, and B) zoomed view of the selected *m/z* region of 700-900 displaying the change in isotope distribution.

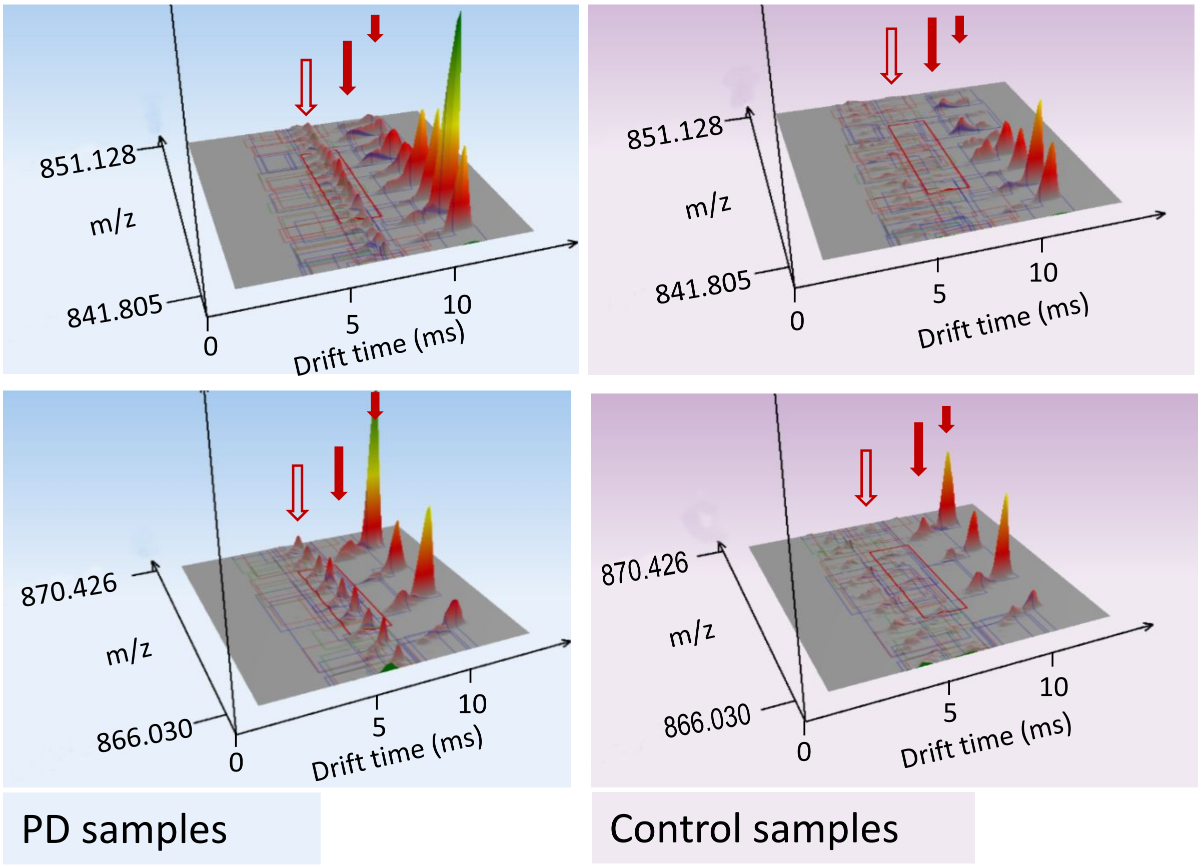

Figure S6. Three-dimensional DT *vs*. *m/z* and intensity distributions for PD (*n*=79) (blue) and control (*n*=71) (magenta) samples from other regions in the DT M/z plots of statistical significance from m/z 840-880. The hollow arrow indicates the region, drift time ~6.7 ms, where there are a series of doubly charged ions that are only present with significant intensity in the PD samples. The two solid arrows indicate the later arriving singly charged species that also present with significantly different intensities between PD and control.

lipid*	cluster	log fold change#	confidence interval†	PrΔ	p‡
TG 42:1	4<br>5	-0.39<br>-0.38	1.75<br>0.14	0.33<br>0	--<br>0.27
TG 43:1	4<br>5	C<br>2.21	--<br>0.25	--<br>1	--<br>0.19
TG 44:1	5	2.78	0.18	1	0.00
TG 45:1	4<br>5	C<br>2.77	--<br>0.26	--<br>1	--<br>0.01
TG 46:1	5	1.33	0.20	1	0.01
TG 47:1	4<br>5	C<br>2.53	--<br>0.25	--<br>1	--<br>0.09
TG 48:1	5	1.08	0.11	1	0.05
TG 49:1	4<br>5	C<br>C	--<br>--	--<br>--	--<br>--
TG 50:1	5	-3.77	3.32	0.04	--
TG 52:1	4<br>5	PD<br>C	--<br>--	--<br>--	--<br>--
TG 53:1	4	C	--	--	--
TG 54:3	5	-2.85	0.28	0	0.22

* tentative identification/annotation; # PD (Parkinson Disease) : C (control); † 95% confidence interval on the log ratio; Δ probability of upregulation (0.95 - 1 significantly up-regulated; 0 - 0.05 significantly down-regulated); ‡ two-tailed T-test; -- = quantitative result cannot be expressed and/or calculated.

Table 3. Differential expression of conformation/configuration clusters of identified lipids by means of PS-IM-MS.

lipid	log fold change#	confidence interval†	Pr∆	p‡
768.6 - [M+A+B]++	0.21	0.56	0.79	0.84
775.1 - [M+A+B]++	0.44	0.81	0.85	0.29
782.1 - [M+A+B]++	1.68	0.29	1	0.03
804.7 - [M+A+B]++	2.47	0.39	1	0.00
810.2 - [M+A+B]++	2.62	0.41	1	0.00
823.2 - [M+A+B]++	2.47	0.31	1	0.00
836.7 - [M+A+B]++	2.47	1.33	1	0.01
843.7 - [M+A+B]++	2.11	0.48	1	0.03
851.2 - [M+A+B]++	0.14	0.28	0.82	0.02
859.2 - [M+A+B]++	1.98	0.47	1	0.41

† 95% confidence interval on the log ratio; ∆ probability of upregulation (0.95 - 1 significantly up-regulated; 0 - 0.05 significantly down-regulated); ‡ two-tailed T-test; -- = quantitative result cannot be expressed and/or calculated; A and B are variable adducts including H, Na, or K, *etc*.

Table 4. Differential expression of doubly charged conformation/configuration (Cluster 3) of lipids detected by means of PS-IM-MS.

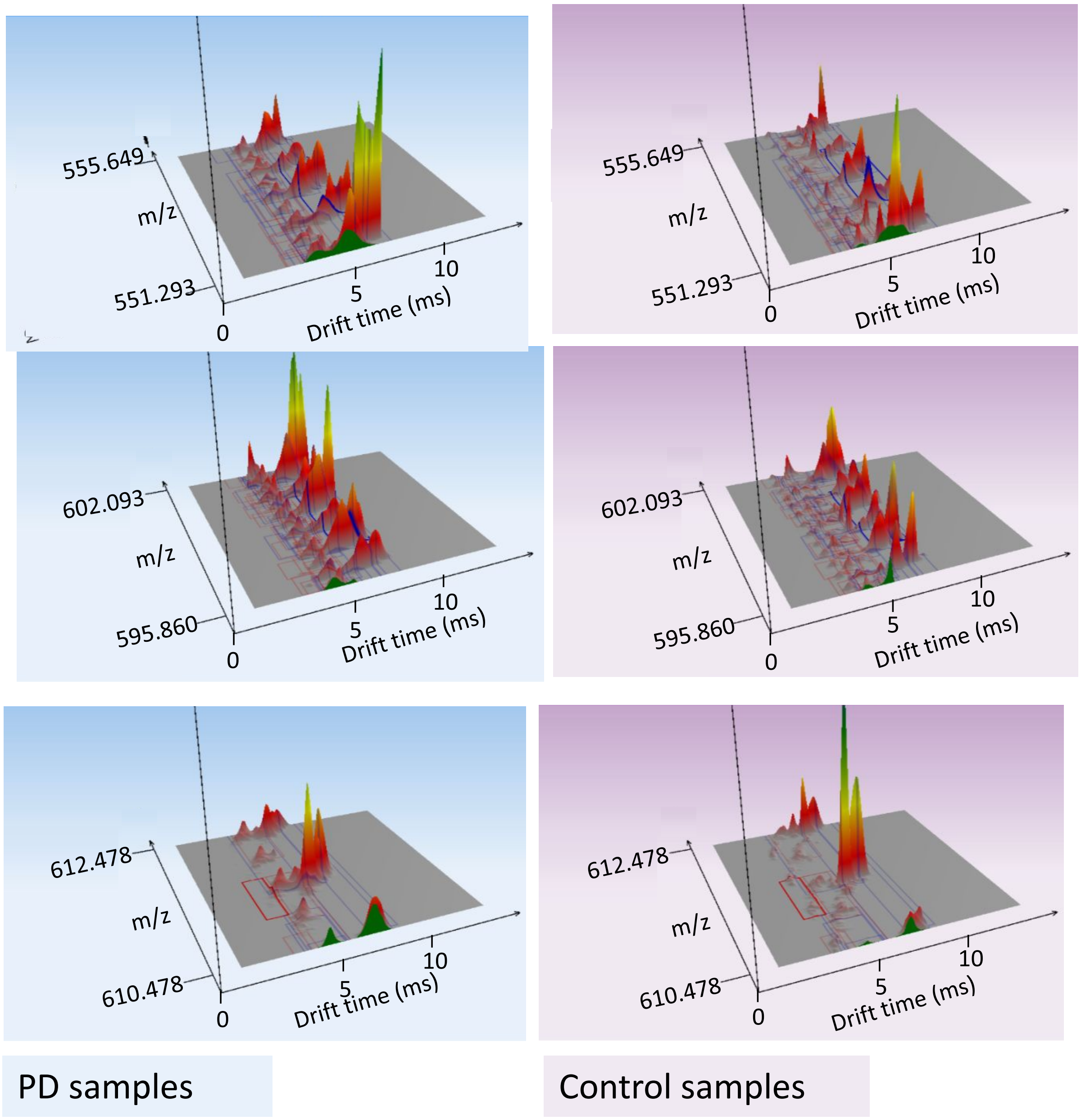

Figure S7. Three-dimensional DT *vs. m/z* and intensity distributions for PD (*n*=79) (blue) and control (*n*=71) (magenta) samples for regions of statistical significance from *m/z* 500-650.

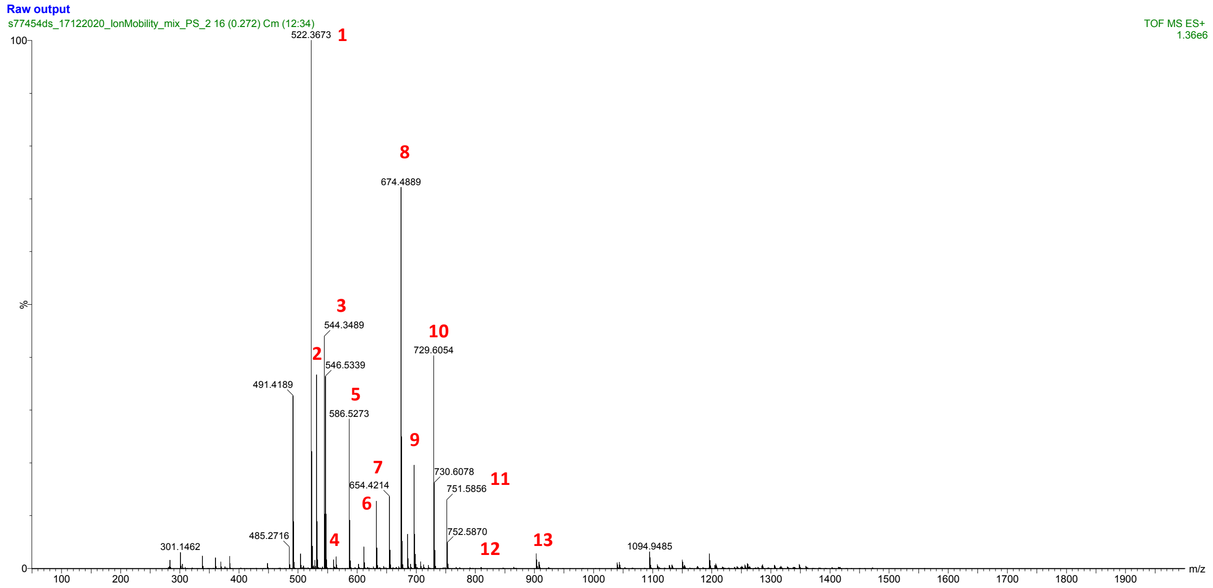

Figure S8. A mass spectrum collected of the standard lipid mixture LightSPLASH™ LIPIDOMIX® Quantitative Mass Spec Primary Standard (Avanti Polar Lipids, Inc., USA), numbers refer to different classes of lipids present in the mix (please refer to Figure 3A in main text for the identities of the numbered ions).

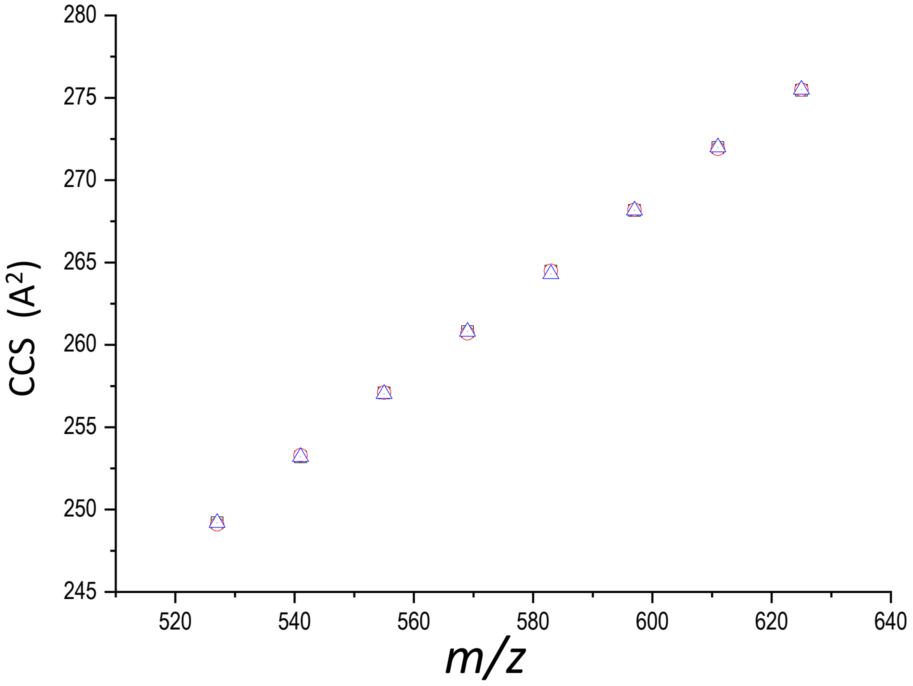

Figure S9. Plots that show $^{TW}CCS_{N2}$ values *vs. m/z* for ions found in sebum in the *m/z* region 500-650.

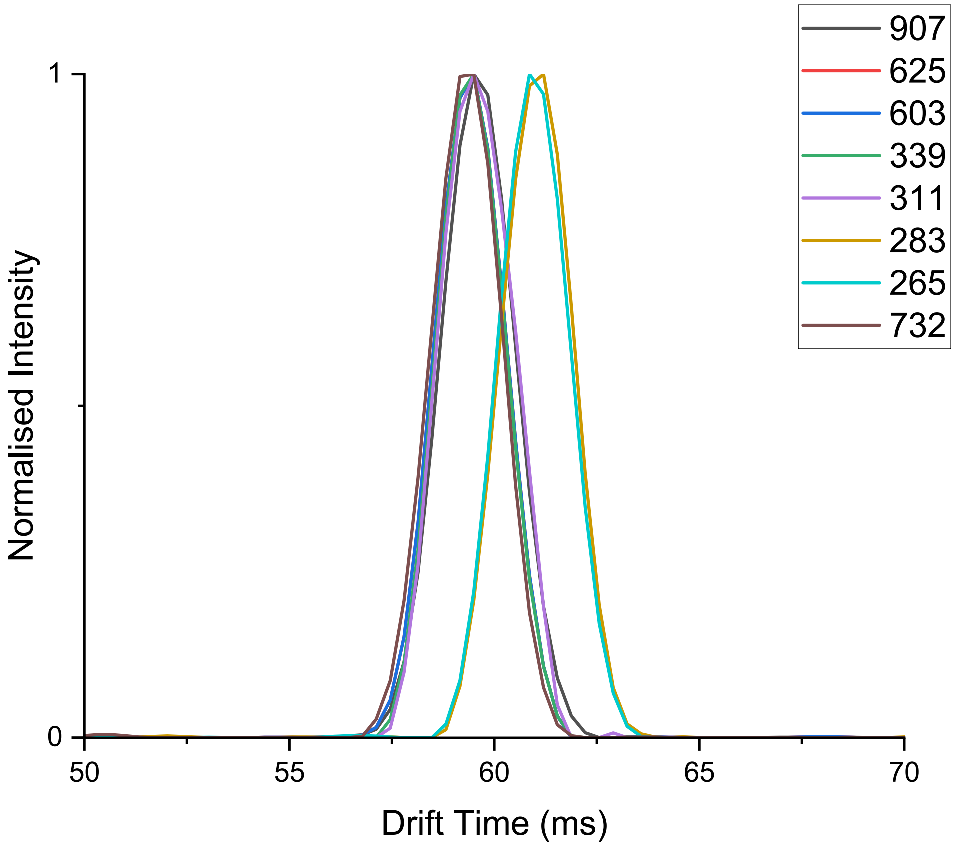

Figure S10A. Extracted ATDs of precursor and dominant fragment ions in sebum (following mass selection of 907; CE 60 V).

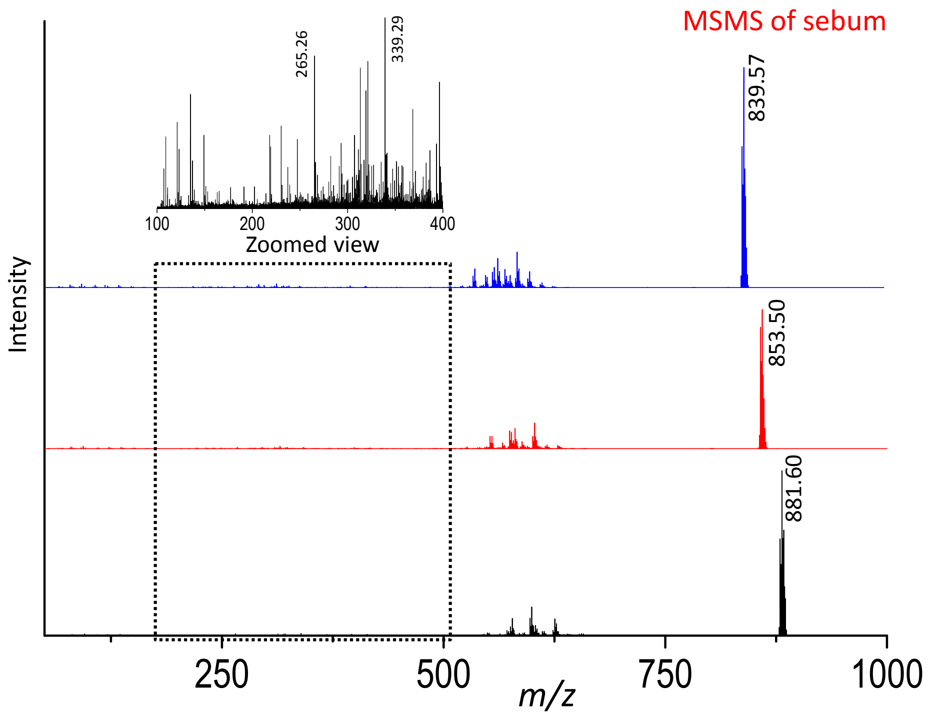

Figure S10B. MSMS spectra from different precursor ions present in sebum, denoted by the mass values RHS occurring at 14 m/z intervals wrt x axis. The inset shows a zoomed view of the spectrum from *m/z* 100-400. Fragment ions at *m/z* 339.29, and 265.26 are common in all these spectra. Both spectra were collected using a collision energy of 30 eV (manufacturer's unit).

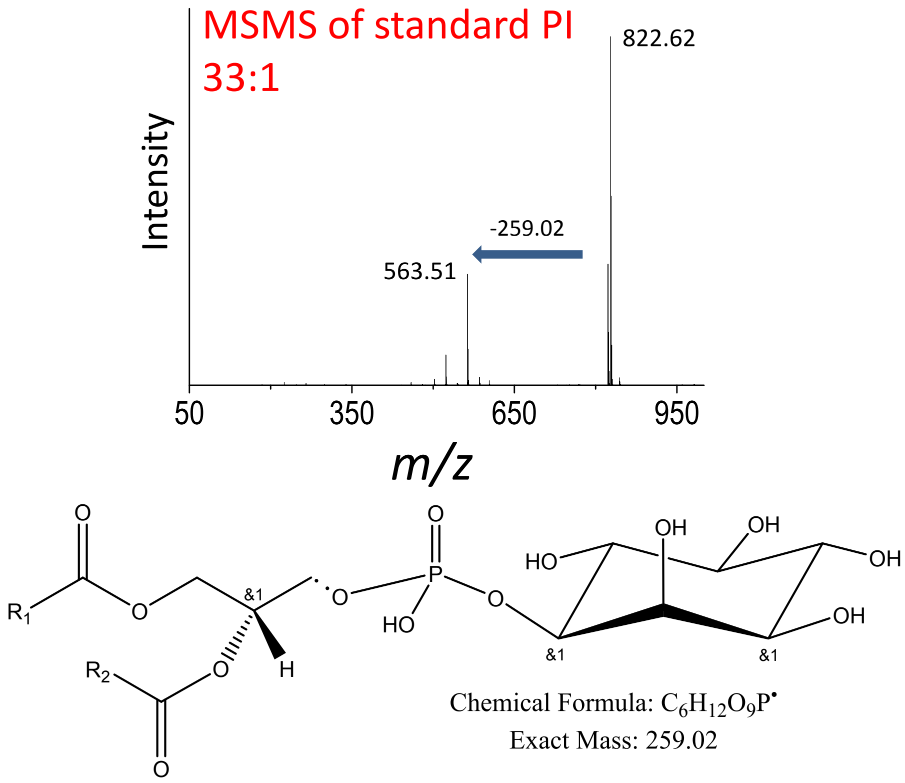

Figure S11. MSMS of PI 33:1 showing a characteristic loss of 259 Da of the polar head group of the PI lipid class. Both spectra were collected using a collision energy of 30 eV (manufacturer's unit).

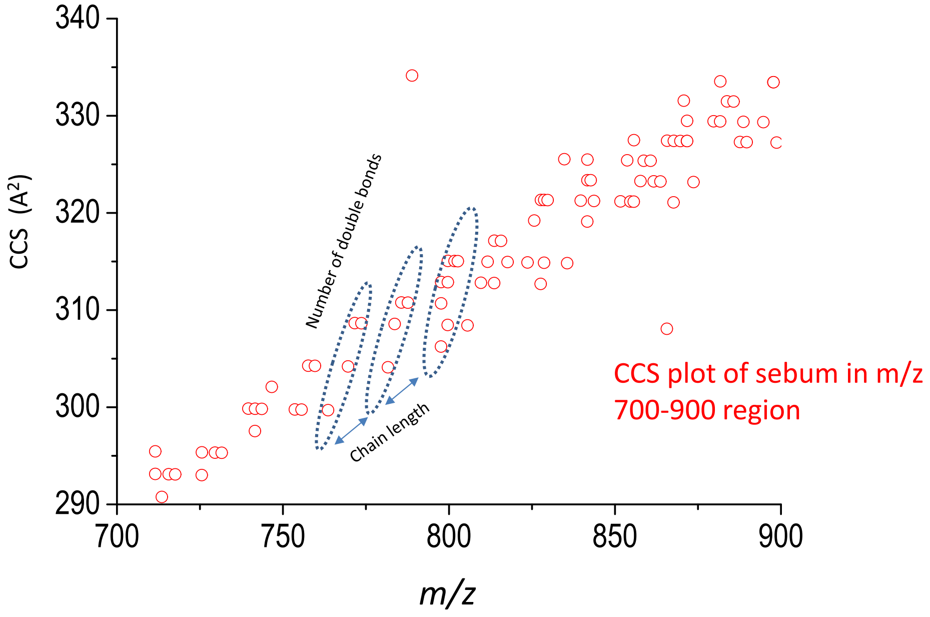

Figure S12. CCS value *vs. m/z* for the lipids present in the mass range 700-900 Da.

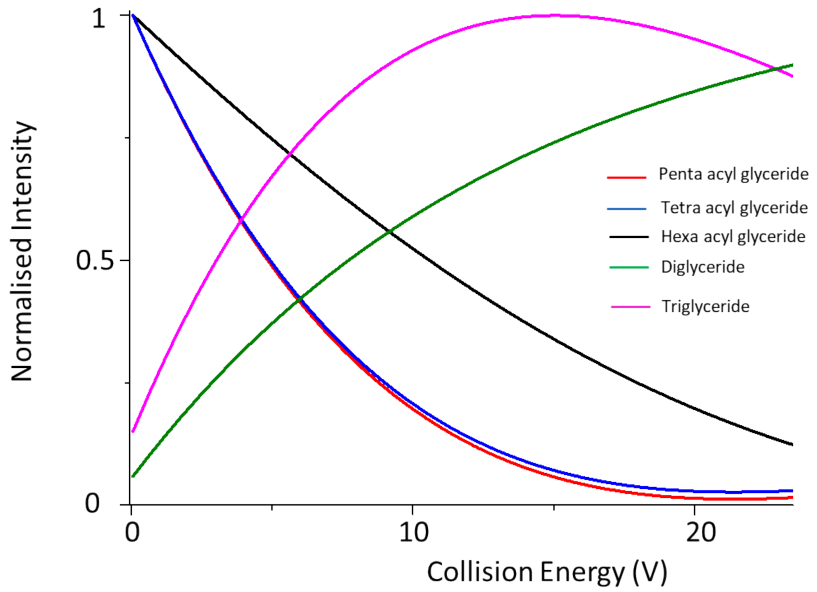

Figure S13. Extracted ion breakdown curves for Penta-acyl glyceride, Tetra-acyl glyceride, Hexa-acyl glyceride, Diglyceride, and Triglyceride with increasing collision energy. The data for this TIC was normalised from data where the collision energy was ramped and then fitted to polynomial curves using Origin Lab21.

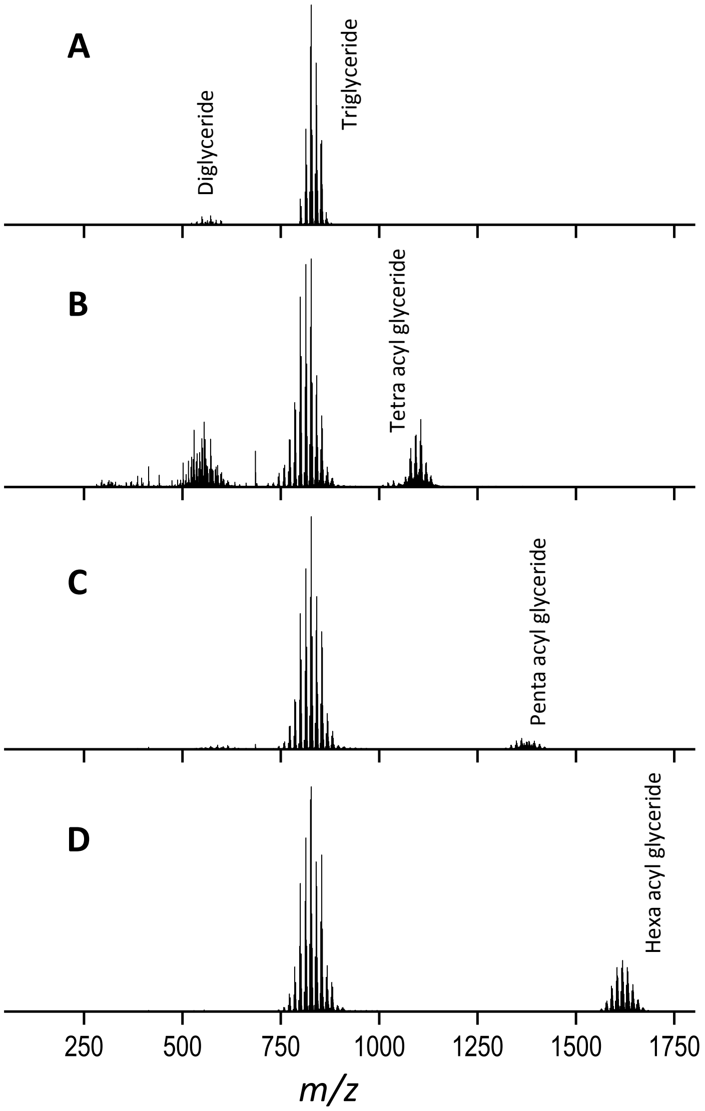

Figure S14. MSMS spectra of selected precursor ions from different regions in the sebum spectrum A) triglyceride, B) tetra-acyl glyceride, C) penta-acyl glyceride, and D) hexa-acyl glyceride.

Parameters	PD	Control
n	79	71
Age (years)[b]	68.83±8.21	63.64±10.38
BMI (kg/m^2)[a,b]	26.78±4.89	27.21±4.89
Gender (Male:Female)[c]	3.15	0.54
Alcohol Intake (Yes:No)[d]	2.03	1.95
Smoker (Yes:No)[d]	0.04	0.04

[a] Body Mass Index (BMI)
[b] BMI and Age values are expressed as mean ± standard deviation
[c] Expressed as a ratio (Male:Female)
[d] Expressed as a ratio (Yes:No)

Table 5. Demographics of participants included in data analysis.

	PD-Control	Significance
Age[a]	$8.5X10^{-4}$	Yes
BMI (kg/m^2)[a]	0.582	No
Gender[b]	$3.31X10^{-10}$	Yes
Alcohol Intake[b]	0.908	No
Smoker[b]	0.894	No

[a] Mann Whitney non-parametric U test used to determine significance, *p*-values were calculated at the 0.05 confidence level
[b] Chi-squared test implemented for categorical variables

Table 6. Statistical significance between PD and control cohorts. The effect of age on metabolism is one of the major confounding factors in many metabolomics studies and we note that we have a statistical variation in age between our controls and PD participants. However, sebum is not a commonly studied biofluid so we would hypothesise that whilst altered lipid metabolism correlating to age has been established in alternative biological matrices, it does not translate directly to metabolites in sebum. We are here measuring metabolites as a secretion output on the skin and hypothesize that these may not mirror the same dysregulation as a function of age that has been reported for other biofluids. Indeed, it has been shown that metabolic age and chronological age are not identical.[1] Our data show that measured lipids that are differential in classification of PD are in fact not influenced by age. This is detailed in our prior publication[2]. The gender matching is also not optimum but is a feature of PD which is a disease that is biased to males, and unfortunately elderly males are less likely to act as control volunteers. We have previously shown, with PLSDA models, that gender is not a distinguishing factor in the metabolites that we analyse from sebum.[2]

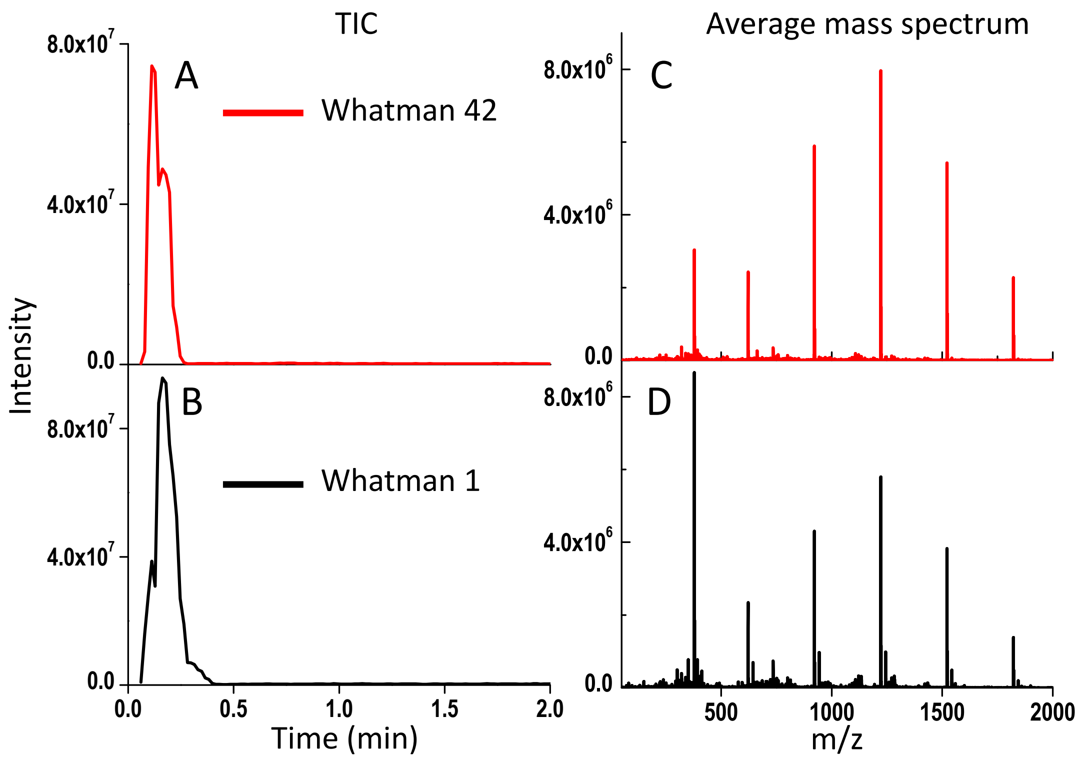

Figure S15. A-B) Total ion chromatogram (TIC) of tune mix (Agilent) using PS-MS from Whatman 42 and Whatman 1 filter papers, respectively. C-D) Corresponding average mass spectra. The TIC and the mass spectra acquired using each filter paper were visually similar, although reproducibility was higher using the Whatman grade 42 paper.

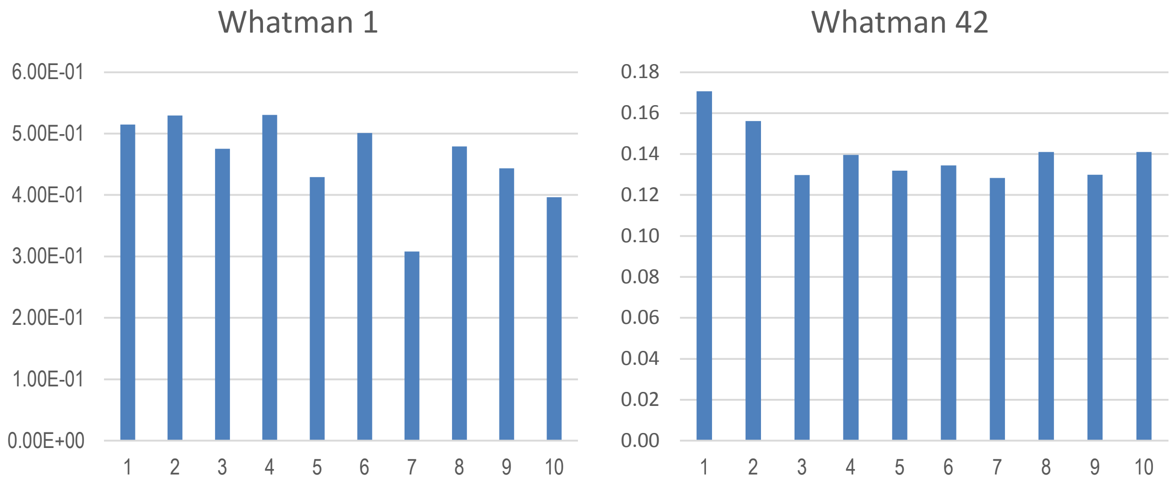

Figure S16. Reproducibility test using a set of 10 samples (L-glutamine under identical conditions), from Whatman 1 and 42 filter papers.

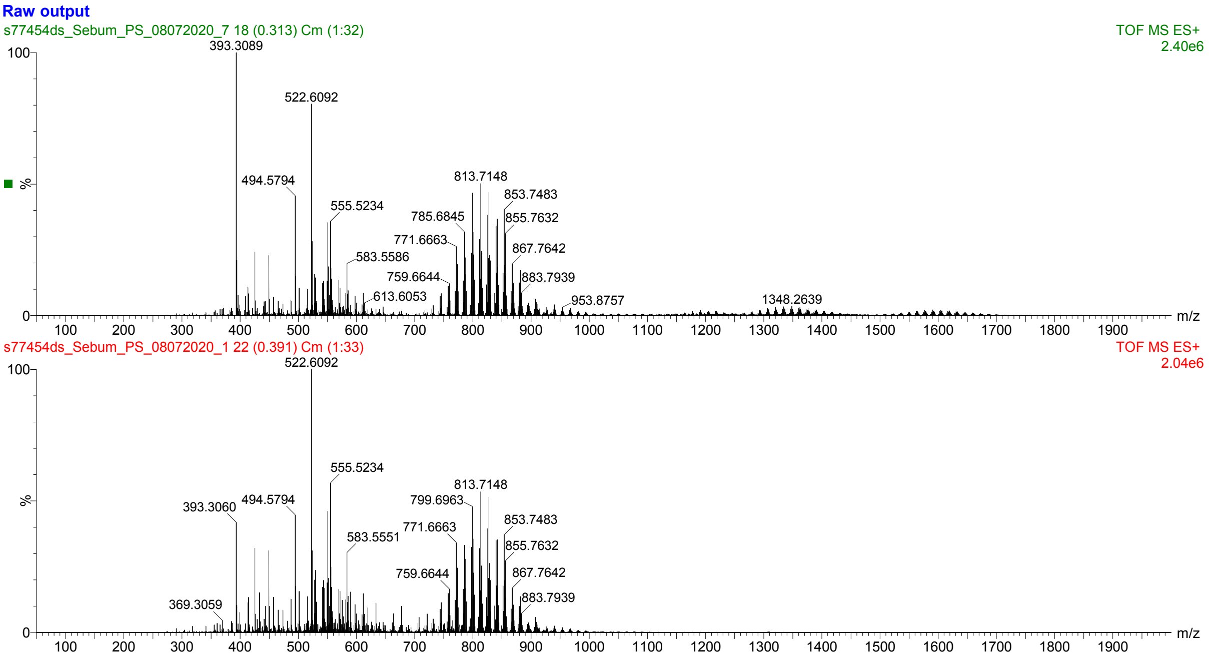

Figure S17. PS-MS mass spectrum of sebum from the same individual recorded at bottom 0h and top 8h intervals.

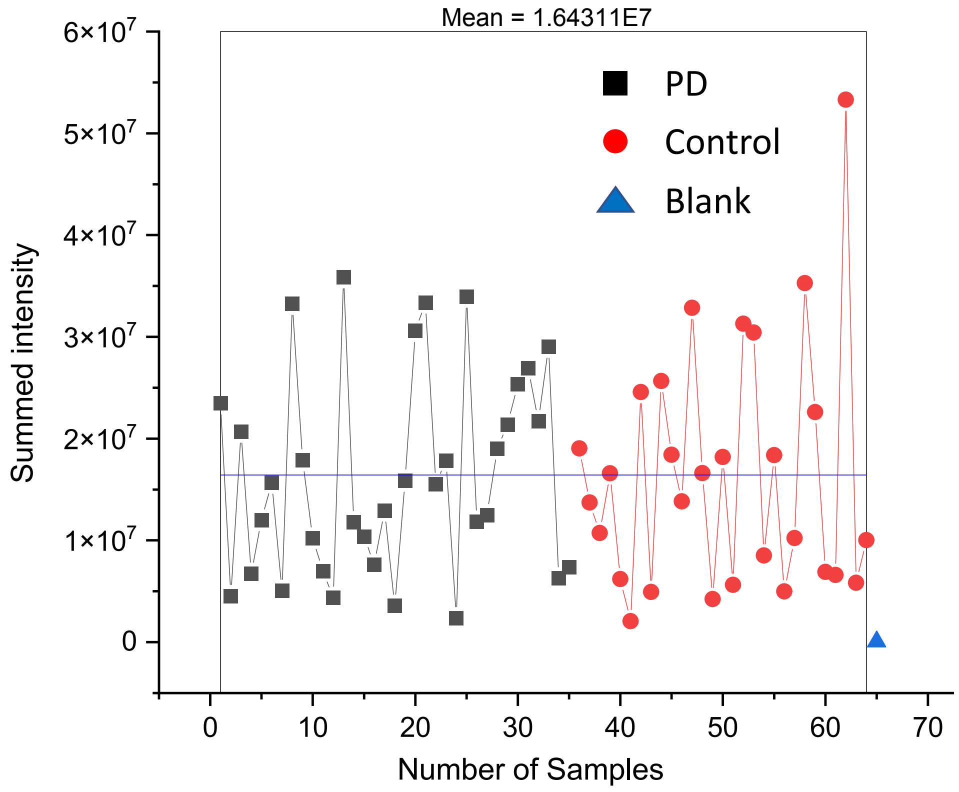

Figure S18. Plot of summed intensities (64 samples) of the features picked by Progenesis QI. Black: PD samples, red: control samples, and blue: blank paper.

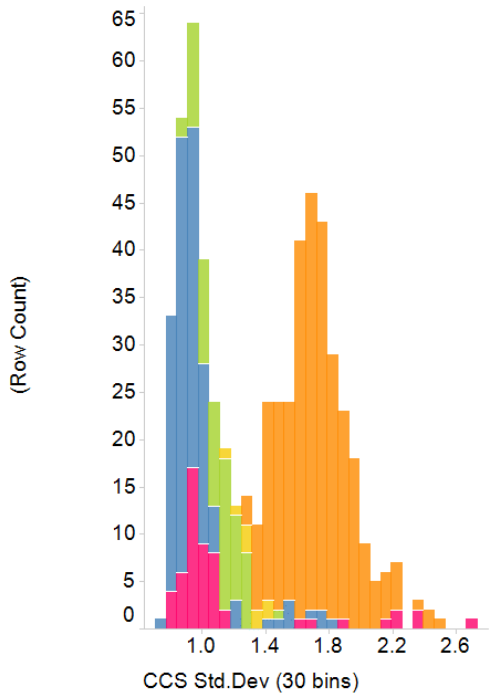

Figure S19. The CCS data were calibrated using a recently reported approach, which gives improved performance for multiply-charged analytes in TWIM devices.[3] CCS calibration uncertainty. Colour = cluster index (red = 1; blue = 2; green = 3; yellow = 4; orange = 5).

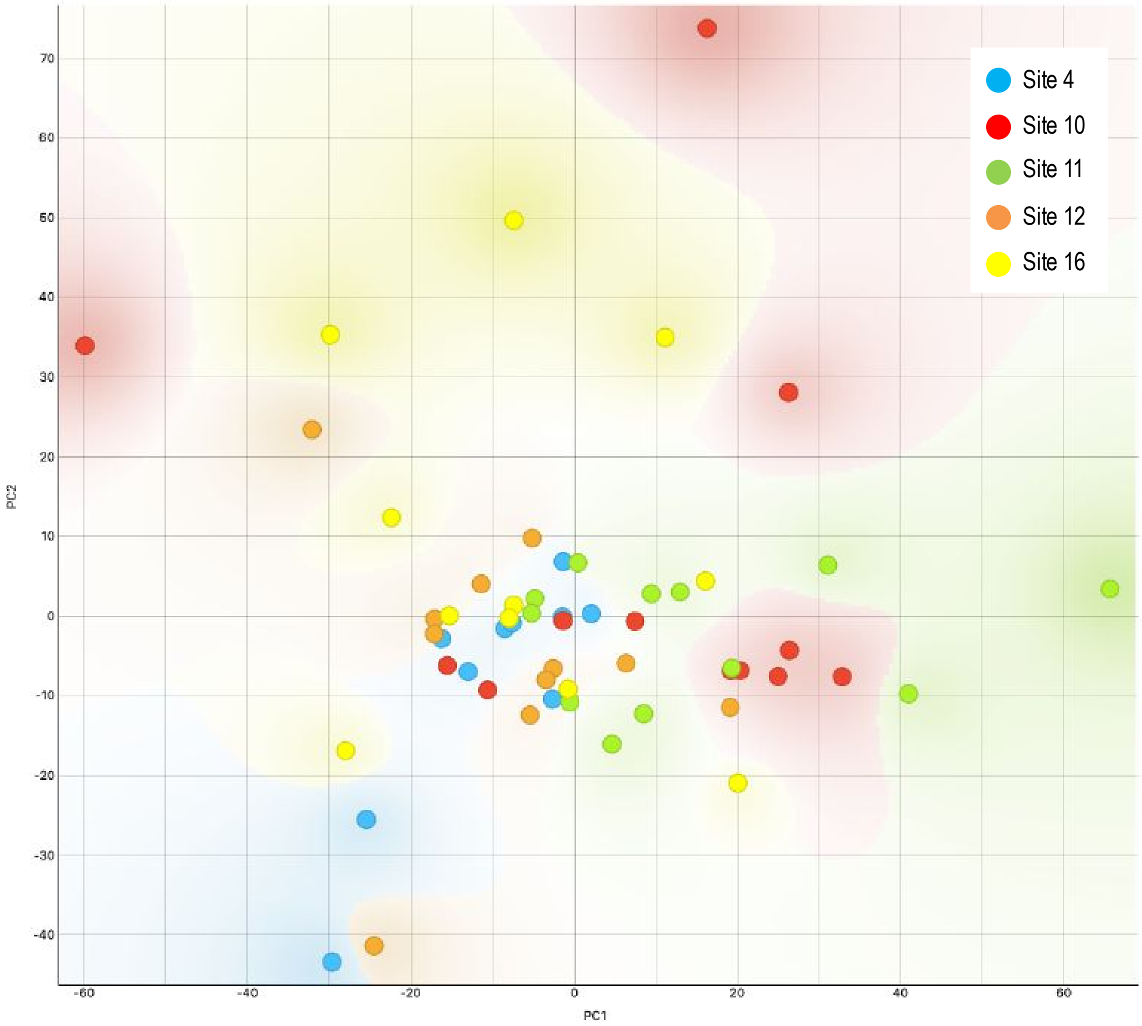

Figure S20. Principal component analysis for PD samples from 5 different recruitment sites (10 samples from each site). The lack of clustering indicates that there is no bias to a given site in the method.

SVM 61%	Predicted Site 4	Predicted Site 10	Predicted Site 11	Predicted Site 12	Predicted Site 15
Actual Site 4	73%	4%	0%	20%	3%
Actual Site 10	7%	23%	25%	27%	18%
Actual Site 11	6%	20%	30%	28%	16%
Actual Site 12	3%	8%	18%	43%	27%
Actual Site 15	2%	16%	11%	48%	23%

Table 7. Confusion matrix using a support vector machine (SVM) model reporting the average prediction output of classifying samples based on collection site (n=100).

RF 30%	Predicted Site 4	Predicted Site 10	Predicted Site 11	Predicted Site 12	Predicted Site 15
Actual Site 4	65%	5%	10%	12%	8%
Actual Site 10	9%	50%	32%	12%	19%
Actual Site 11	10%	35%	23%	20%	13%
Actual Site 12	15%	21%	23%	24%	17%
Actual Site 15	16%	25%	21%	25%	13%

Table 8. Confusion matrix using a random forest (RF) model reporting the average prediction output of classifying samples based on collection site (*n*=100).

References:

(1) Rattray, N. J. W.; Trivedi, D. K.; Xu, Y.; Chandola, T.; Johnson, C. H.; Marshall, A. D.; Mekli, K.; Rattray, Z.; Tampubolon, G.; Vanhoutte, B.; White, I. R.; Wu, F. C. W.; Pendleton, N.; Nazroo, J.; Goodacre, R. Metabolic Dysregulation in Vitamin E and Carnitine Shuttle Energy Mechanisms Associate with Human Frailty. *Nat. Commun. 2019 101* **2019**, *10* (1), 1–12. https://doi.org/10.1038/s41467-019-12716-2.

(2) Sinclair, E.; Trivedi, D. K.; Sarkar, D.; Walton-Doyle, C.; Milne, J.; Kunath, T.; Rijs, A. M.; de Bie, R. M. A.; Goodacre, R.; Silverdale, M.; Barran, P. Metabolomics of Sebum Reveals Lipid Dysregulation in Parkinson's Disease. *Nat. Commun.* **2021**, *12* (1). https://doi.org/10.1038/s41467-021-21669-4.

(3) Richardson, K.; Langridge, D.; Dixit, S. M.; Ruotolo, B. T. An Improved Calibration Approach for Traveling Wave Ion Mobility Spectrometry: Robust, High-Precision Collision Cross Sections. *Anal. Chem. (Washington, DC, United States)* **2021**, *93* (7), 3542–3550. https://doi.org/10.1021/acs.analchem.0c04948.
